# Zinc Finger Nuclease Mediated Knockout of ADP-Dependent Glucokinase in Cancer Cell Lines: Effects on Cell Survival and Mitochondrial Oxidative Metabolism

**DOI:** 10.1371/journal.pone.0065267

**Published:** 2013-06-14

**Authors:** Susan Richter, Shona Morrison, Tim Connor, Jiechuang Su, Cristin G. Print, Ron S. Ronimus, Sean L. McGee, William R. Wilson

**Affiliations:** 1 Auckland Cancer Society Research Centre, The University of Auckland, Auckland, New Zealand; 2 Department of Molecular Medicine and Pathology, Faculty of Medical and Health Sciences, The University of Auckland, Auckland, New Zealand; 3 The New Zealand Bioinformatics Institute, The University of Auckland, Auckland, New Zealand; 4 Metabolic Research Unit, School of Medicine, Deakin University, Geelong, Victoria, Australia; 5 AgResearch, Palmerston North, New Zealand; Center for Genomic Regulation, Spain

## Abstract

Zinc finger nucleases (ZFN) are powerful tools for editing genes in cells. Here we use ZFNs to interrogate the biological function of *ADPGK*, which encodes an ADP-dependent glucokinase (ADPGK), in human tumour cell lines. The hypothesis we tested is that ADPGK utilises ADP to phosphorylate glucose under conditions where ATP becomes limiting, such as hypoxia. We characterised two ZFN knockout clones in each of two lines (H460 and HCT116). All four clones had frameshift mutations in all alleles at the target site in exon 1 of *ADPGK,* and were ADPGK-null by immunoblotting. *ADPGK* knockout had little or no effect on cell proliferation, but compromised the ability of H460 cells to survive siRNA silencing of hexokinase-2 under oxic conditions, with clonogenic survival falling from 21±3% for the parental line to 6.4±0.8% (p = 0.002) and 4.3±0.8% (p = 0.001) for the two knockouts. A similar increased sensitivity to clonogenic cell killing was observed under anoxia. No such changes were found when *ADPGK* was knocked out in HCT116 cells, for which the parental line was less sensitive than H460 to anoxia and to hexokinase-2 silencing. While knockout of *ADPGK* in HCT116 cells caused few changes in global gene expression, knockout of *ADPGK* in H460 cells caused notable up-regulation of mRNAs encoding cell adhesion proteins. Surprisingly, we could discern no consistent effect on glycolysis as measured by glucose consumption or lactate formation under anoxia, or extracellular acidification rate (Seahorse XF analyser) under oxic conditions in a variety of media. However, oxygen consumption rates were generally lower in the *ADPGK* knockouts, in some cases markedly so. Collectively, the results demonstrate that *ADPGK* can contribute to tumour cell survival under conditions of high glycolytic dependence, but the phenotype resulting from knockout of *ADPGK* is cell line dependent and appears to be unrelated to priming of glycolysis in these lines.

## Introduction

The identification of large numbers of candidate genes through genomic analysis has created a pressing need for new approaches for ascribing biological function. Highly sequence-specific zinc-finger nucleases (ZFN) have utility for targeted gene editing in live cells [1]–[6] and are one of the emerging functional genomics tools for exploring genotype/phenotype relationships. Specifically, dimeric ZFNs capable of recognising 18–42 base pair sequences can be used to introduce double strand DNA breaks at unique locations in the genome. These DNA breaks initiate error-prone non-homologous end joining repair to generate site-specific, heterogeneous mutations (predominantly small indels that disrupt gene function) or, in the presence of a donor DNA sequence, to introduce defined mutations via homology-directed repair. Recent studies confirm the high sequence specificity of custom-designed ZFNs in cells [7]–[10].

Here we utilise ZFN technology to interrogate the biological function of a human gene, *ADPGK*, which encodes an ADP-dependent glucokinase (ADPGK). Surprisingly, given extensive investigation of glucose phosphorylation as the central reaction of intermediary metabolism for many decades [11], mammalian ADPGK was discovered only recently through its sequence similarly to archaeal ADP-dependent glucokinases [12]. Phylogenetic analysis suggests the ancestral gene was laterally transferred from Archaea early in metazoan evolution [12]. The purified recombinant murine [12] and human [13] enzymes have been confirmed to phosphorylate glucose, with the unusual feature (as for the archaeal enyzmes [14]) that the phosphoryl donor is ADP in contrast to the well-studied ATP-dependent vertebrate hexokinase isoforms (HK1-4). Murine ADPGK is quite specific for glucose, with lesser ability to phosphorylate mannose and fructose (20% and 10% respectively of the rate with glucose); it has a low apparent K_M_ for both glucose (96 µM) and ADP (260 µM), and is inhibited by high concentrations of glucose and by its product AMP but, unlike HK1-4, not by glucose-6-phosphate. The biological role of eukaryotic ADPGKs is not well understood; RNAi screens in *C. elegans* and HeLa cells have not identified a phenotype [15]–[17], although a recent report has demonstrated a role of ADPGK in T-cell receptor signalling through diversion of glycolytic flux to the glycerol-3-phosphate dehydrogenase shuttle, resulting in stimulation of mitochondrial production of reactive oxygen species (ROS) [18]. However, the biochemical properties of ADPGK, particularly its ability to utilise ADP, led us to hypothesise that it may play a role in priming glycolysis under stress conditions where ATP becomes limiting [12], such as under hypoxia when cells become highly dependent on glycolytic ATP generation [19].

Given the importance of glucose phosphorylation in tumour metabolism, we focus here on the role of ADPGK in human tumour cell lines. Tumour cells are highly dependent on glycolysis, as first observed by Otto Warburg during the 1920s [20], [21], and the associated metabolic reprogramming has recently been suggested as a possible hallmark of cancer [22]. In particular, elevated glycolytic flux in tumour cells is considered to provide intermediates for anabolic pathways and to increase antioxidant defenses through NADPH generation via the pentose phosphate pathway [23], [24]. Expression of hexokinases is often up-regulated in cancer cells as part of this metabolic switch [25]. ADPGK is highly expressed in human tumours and tumour cell lines, at both the mRNA and protein levels, although there is little indication of up-regulation relative to normal tissues and (unlike many glycolytic enzymes) it is not up-regulated by anoxia, hypoxia or HIF-1 in tumour cell cultures and shows little dependence on extracellular glucose concentration [13]. However, given that an emergency response to ATP depletion would be mounted most rapidly by a constitutively expressed enzyme, the lack of regulation of *ADPGK* expression by hypoxia does not preclude a role in priming glycolysis under these conditions.

Our initial attempt to test this hypothesis examined the effect of suppressing *ADPGK* expression, with and without suppression of HK2, in HCT116 and H460 human tumour cell lines using RNA interference [13]. This study showed higher mRNA and protein expression of both ADPGK and HK2 in H460 than in HCT116 cells, but did not demonstrate any significant effect on anaerobic glycolysis (glucose consumption and lactate formation), or on clonogenic cell survival under short term anoxia in either line, although a small decrease in aerobic plating efficiency of H460 cells was shown when *ADPGK* was knocked down [13]. However, inhibition of *ADPGK* expression was incomplete in this study (typically ∼60–90% reduction in protein indicated by western blotting), raising the question as to whether residual ADPGK activity may have masked the phenotype.

In the present study we circumvent the incomplete silencing of *ADPGK* by using specific ZFNs to effect multi-allelic mutational inactivation of the gene, generating ADPGK-null H460 and HCT116 cell lines. The pair of ZFNs used here target a genomically unique 37 base pair recognition sequence within the first exon of the *ADPGK* gene. Two independent *ADPGK*-null clones with confirmed frameshift mutations were generated in each genetic background. These clones were characterised with respect to proliferation, clonogenic survival and glycolytic flux under aerobic and anaerobic conditions. Extracellular acidification (as a measure of glycolysis) and mitochondrial oxygen consumption were also measured using a Seahorse XF analyser. Finally, the ability of these cell lines to grow as tumour xenografts, and the steady state proportion of hypoxic cells in the resulting tumours, were compared with parental lines.

## Results

### Generation of *ADPGK*-null Cell Lines using ZFNs

**Figure 1:**
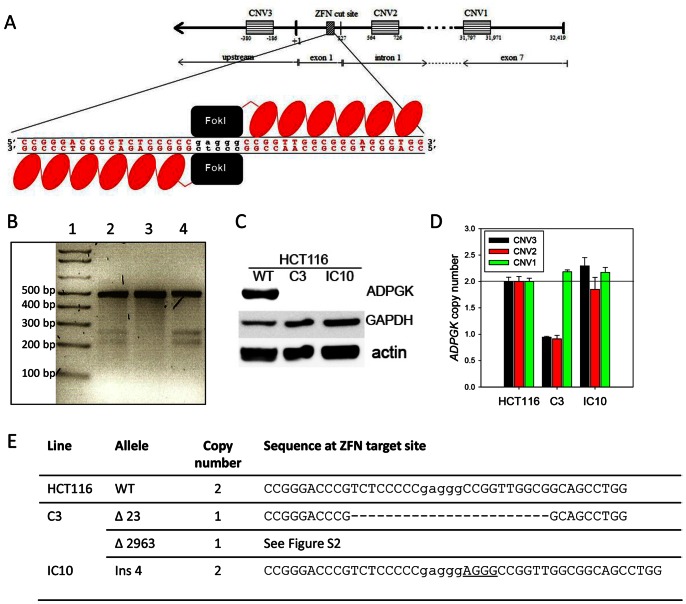
*APDGK* knockout in HCT116 cells using zinc-finger nucleases. (A) Features of the *ADPGK* gene, with location of primer pairs and the dimeric ZFN targeting site. (B) PCR/CEL-II nuclease (Surveyor) assay for mutation detection at the ZFN target site. HCT116 cells were transfected with a pair of ADPGK ZFNs and a GFP plasmid. One day later genomic DNA was prepared from pooled FACS sorted GFP positive cells for the assay (Lane 4). Lane 1: DNA ladder. Lane 2: positive control DNA from ADPGK ZFN-treated K562 cells. Lane 3: Untreated HCT116 cells. (C) ADPGK ZFN-treated HCT116 cells cloned and screened for *ADPGK* by western blot. (D) Genomic DNA copy number by qPCR for primer pairs CNV1-3. Results are plotted relative to WT DNA which has two *ADPGK* alleles. (E) Sequencing across ZFN recognition site (predicted cut site in lower case) resulted in deletions (−) and insertions (underlined) for *ADPGK*-null HCT116 clones C3 and IC10. The inferred copy number for each allele is shown.

*ADPGK* was knocked out in two genetic backgrounds (HCT116 and H460) using CompoZr™ ZFNs custom-designed to introduce double-strand breaks at a genomically unique target site in the first exon of the *ADPGK* gene (Figure 1A). The ability of the ZFNs to introduce mutations at this site was tested in HCT116 cells using the Surveyor™ mutation detection assay (Figure 1B), following lipid-based co-transfection with a GFP plasmid and FACS sorting 24 h later to enrich for transfected cells. A 473 bp region surrounding the ZFN cut site was amplified by PCR and the products were denatured and re-annealed to generate mismatch bubbles at ZFN cut sites. CEL-II nuclease [26] cleavage of the mismatch bubbles resulted in the predicted products (256 and 217 bp) for cleavage at the target site. Band densitometry of the image in Figure 1B showed that the ratio of the summed cleavage products to parental band was 66% (0.399/0.601) for the ZFN-treated DNA supplied by the manufacturer (lane 2) and 68% (0.403/0.597) for ZFN-transfected HCT116 cells (lane 4) in this experiment, indicating a high frequency of site-specific mutations. In later experiments, electroporation was used instead of lipid-based transfection to further increase transfection efficiencies.

Clones isolated from *ADPGK* ZFN-treated populations were screened by immunoblotting for the absence of the characteristic 54-kDa ADPGK band. Two candidate knockouts (HCT116 C3 and IC10) with no detectable ADPGK protein (Figure 1C) were identified from two different rounds of transfection (C3 from screening of 12 clones after FACS enrichment, and IC10 from 131 clones after electroporation without flow sorting using conditions which gave transfection efficiencies of ∼70% using a GFP plasmid), indicating a low overall frequency of bi-allelic knockout leading to complete loss of immunodetectable protein. A higher proportion of clones appeared to show reduced ADPGK protein expression (data not shown), possibly due to mutational inactivation of a single allele. To test whether the low frequency of null lines (2/143 clones) reflects compromised survival or proliferation in bi-allelic knockouts, we followed the frequency of mutations in a ZFN-transfected HCT116 pool using the Surveyor (PCR/CEL-II nuclease) assay. The 256 and 217 bp bands diminished over two weeks in culture (Figure S1A), although a similar decrease over time was seen with a separate ZFN pair targeting the 8^th^ exon of *POR* (Figure S1B), which encodes NADPH;cytochrome P450 oxidoreductase. POR-null HCT116 clones were isolated at higher frequency (3/14) using the *POR* ZFN pair. Thus the progressive loss of *ADPGK* and *POR* mutations is more likely to reflect compromised proliferation/survival of cells with the highest plasmid copy number after transfection.

Copy number within the *ADPGK* gene was assessed for the two ADPGK null clones (C3 and IC10) by qPCR using primer pairs flanking the ZFN target sequence (CNV2 and 3) and another recognising exon 7 (CNV1) >31 kb away (Figure 1D). This showed the presence of two copies of *ADPGK* in the knockouts, as for the parent line (consistent with the Sanger Cancer Genome Project; www.sanger.ac.uk/cgi-bin/genetics/CGP/cghviewer/CghViewer.cgi), except for a single copy region near the ZFN target cite in HCT116 clone C3. Sequencing across the ZFN target site of genomic DNA from these null clones demonstrated frameshift mutations in both (Figure 1E). These frameshifts generate premature stop codons resulting in predicted truncated proteins of 75 and 84 amino acids, lacking the ADP kinase domain located between amino acids 52 and 497 of the full length isoform (www.uniprot.org), and are thus expected to be catalytically inactive. The finding of only one mutated (and no WT) sequence for clone 1C10, coupled with a copy number of two at CNV2 and 3, suggests homologous recombination using the initial ZFN-induced mutation as template resulted in reduction to homozygosity. HCT116 C3 also provided only a single sequence at the ZFN target site, but the copy number of one at CNV2 and 3 suggested a large deletion. This was confirmed by amplifying and sequencing an extended region, identifying a 2963 deletion spanning the CNV2 and CNV3 sites (Figure S2).

**Figure 2:**
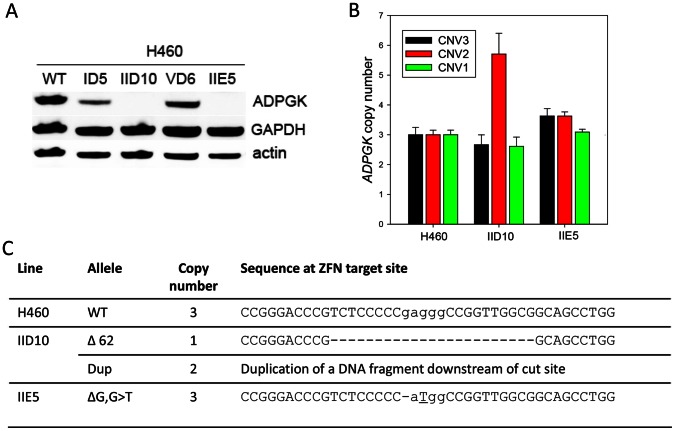
*APDGK* knockout in H460 cells using zinc-finger nucleases. (A) Western blots of H460 cells after transfection with ADPGK ZFNs (clones ID5 and VD6), and *ADPGK*-null clones derived from these in a second round of ZFN transfection (IID10 and IIE5). (B) Genomic DNA copy number by qPCR for primer pairs CNV1-3. Results are plotted relative to WT DNA which has three *ADPGK* alleles. (C) Sequencing across the ZFN cut site shows deletions (−) and insertions (underlined) for the *ADPGK* alleles in H460 clones IID10 and IIE5.

Transfection of H460 cells with the same *ADPGK* ZFN plasmids yielded no *ADPGK*-null clones from screening of 127 clones after nucleofection alone and screening of 24 clones after nucleofection and FACS enrichment. This low efficiency may reflect the presence of three *ADPGK* alleles in H460 (Sanger Cancer Genome Project), which we confirmed with the qPCR-based copy number assay (Figure S3). Two clones showed reduced ADPGK protein levels (ID5 and VD6; Figure 2A), suggesting they may carry mutant alleles, were therefore subjected to a second round of ZFN transfection (electroporation and FACS), providing 5/121 clones with no detectable ADPGK protein from which two were chosen for further analysis (IID10 from ID5 and IIE5 from VD6) (Figure 2A). Copy number determination (Figure 2B) in conjunction with sequencing of the ZFN target site (Figure 2C) showed a homozygous frameshifting compound deletion/base substitution mutation for clone H460 IIE5 giving a predicted truncated protein of 89 amino acids. Clone IID10 had a 62-bp deletion in at least one allele, potentially generating a protein of 62 amino acids, while the other allele(s) carry a number of duplications of a segment of DNA downstream from the ZFN cutting site, which were not fully identified.

### Proliferation and Clonogenicity of *ADPGK* Knockout Cell Lines

**Figure 3:**
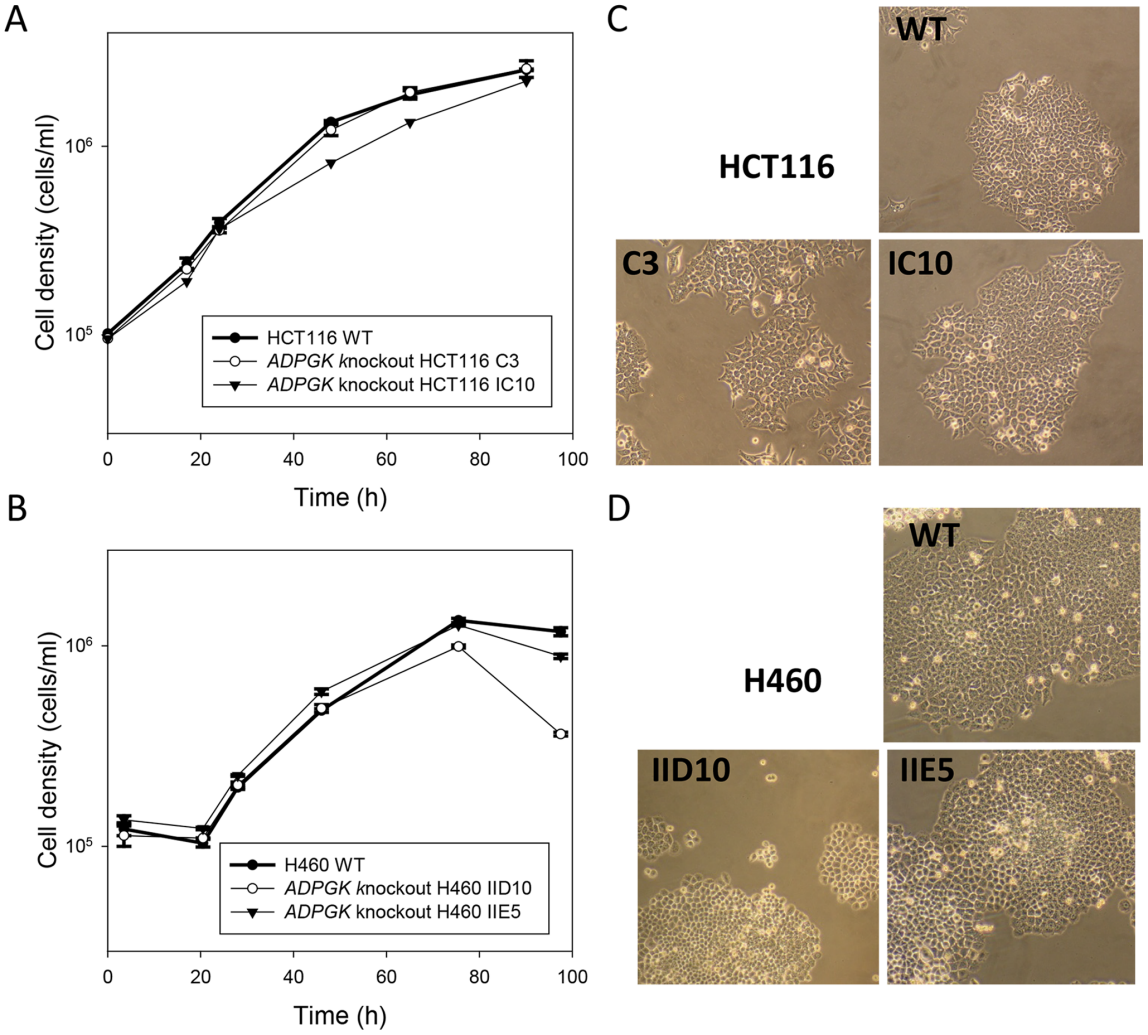
*ADPGK* knockout clones of HCT116 (A, C) and H460 (B, D) show growth and morphology similar to the respective WT lines *in vitro*. (A/B). Cell density after seeding 24-well plates at 10^5^/ml. Values are mean and SEM for triplicate cultures. (C/D). Phase contrast microscopy (10× objective magnification) of WT and knockout clones in T flasks.

The ADPGK-null cell lines from both genetic backgrounds showed similar doubling times to the WT lines when cultured under standard aerobic cell culture conditions (Figure 3A and B), with a possible slight reduction in cell proliferation for the HCT116 IC10 line. Colony morphology by phase contrast microscopy was also similar to the parental cell lines (Figure 3C and D) although the H460 IID10 cells were somewhat more prone to rounding up and detaching from the dish.

To further investigate proliferation, HCT116 and H460 cell lines were cultured under normoxia for 54 and 72 h, respectively, and cell number, cell volume, cell cycle distribution and clonogenicity was measured (Table 1). No significant differences were observed for HCT116 C3 compared to WT; however a two-fold increase in G1 phase cells was seen for HCT116 IC10, consistent with the reduction in proliferation noted above. Plating efficiency was similar for all three HCT116 cell lines, but the slower proliferation of HCT116 IC10 was reflected in a significantly decreased colony radius. Both H460 *ADPGK*-null clones showed a small but significant decrease in cell number, and a small increase in G2/M phase cells. Additionally, H460 IID10 had an increased mean cell volume and showed a trend towards decreased plating efficiency as well as decreased colony radius, possibly due to the detachment of cells from the colonies. Overall, the results suggest a minor effect on proliferation but this was not a consistent finding across cell lines and clones.

**Table 1:** Proliferation and clonogenicity of HCT116 and H460 cell lines under normoxia.

Sample	Fold increase in cell number	Mean cell volume (fl)	Cell cycle			Clonogenic assay	
			G1/G0	S	G2/M	Plating efficiency (%)	Colony radius (mm)
HCT116 (54 h incubation)							
**HCT116 WT**	3.32±0.17	1722±17.8	30.1±0.7	46.7±0.4	22.0±0.4	113.0±5.7	0.91±0.004
**C3**	3.18±0.26	1722±0.3	26.1±0.8	48.3±0.5	24.6±0.5	115.7±4.5	0.92±0.002
**IC10**	3.00±0.38	1676±9.3	58.7±1.0 *	25.6±0.6 *	14.5±0.3 *	96.6±6.3	0.64±0.016 *
H460 (72 h incubation)							
**H460 WT**	12.87±0.10	1814±43.0	60.3±0.8	29.5±1.0	8.8±0.3	92.0±2.3	0.54±0.007
**IIE5**	9.38±0.29 *	1824±36.0	59.4±0.1	28.6±0.1	10.3±0.3 *	92.1±5.9	0.54±0.004
**IID10**	9.44±0.83 *	2338±38.8 *	61.0±0.9	28.0±0.8	10.0±0.2 *	75.9±14.7	0.43±0.007 *

Asterisks indicate significant (p<0.05) difference from WT by one-way ANOVA/Holm-Sidak. Proliferation parameters of respective cell lines are displayed as mean ± SEM for three biological replicates.

### Global Gene Expression in *ADPGK*-null Lines

To investigate whether knockout of *ADPGK* affects gene expression, two *ADPGK* –null clones (HCT116 C3 and H460 IIE5) with similar proliferation and colony morphology to their parental lines were chosen for microarray analysis using Affymetrix Human Gene 1.0 ST microarrays. Single cultures of the HCT116 C3 *ADPGK*-null line and HCT116 parental cells were compared in a first experiment, and duplicate cultures of both the HCT116 and H460 pairs in a second experiment.

**Figure 4:**
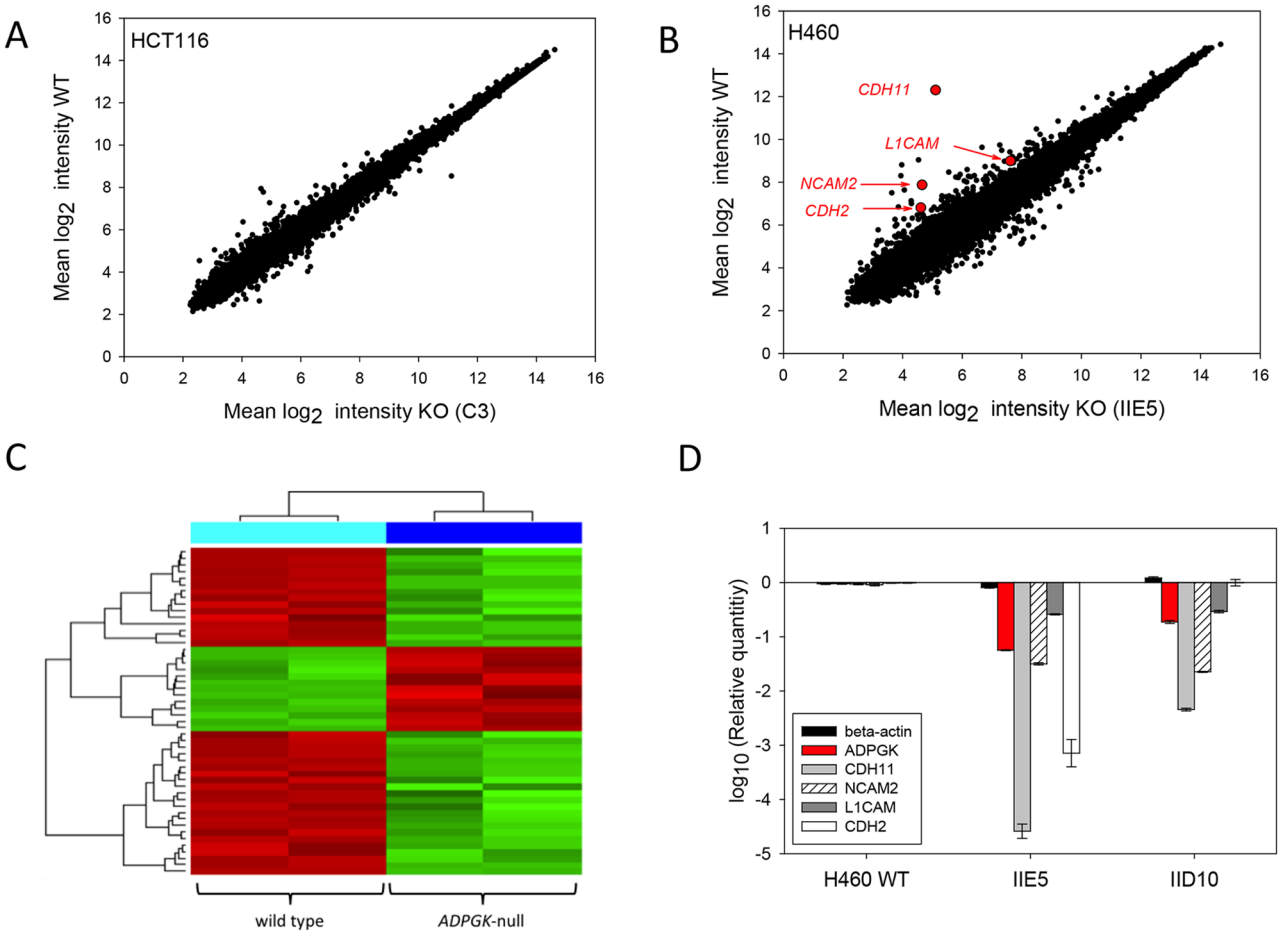
Global gene expression in ADPGK-null HCT116 (clone C3) and H460 (clone IIE5) cell lines. RNA was analyzed using Affymetrix Human Gene 1.0 ST microarrays (A) Scatterplot showing mean expression values for HCT116 C3 versus WT (3 cultures each). (B) Scatterplot showing mean expression values for H460 IIE5 versus WT (2 cultures each). (C) Heat map of the 50 top-ranked differentially expressed probe sets in duplicate cultures of H460 WT and H460 IIE3. (D) qPCR of RNA from 3 replicate cell cultures each for H460 WT and *ADPGK* knockout clones IIE5 and IID10, normalised against 18S rRNA.

The transcriptome of the *ADPGK* –null HCT116 C3 line appeared to be broadly similar to that of the WT HCT116 line (Figure 4A) and no differentially expressed transcripts were identified when the false discovery rate was controlled to 5% by the Benjamini-Hochberg method. While this analysis suggested that the number of apparently differentially expressed transcripts was no greater than expected due to chance, it remains possible that small numbers of mRNA transcripts may be authentically regulated following *ADPGK* gene inactivation in HCT116 cells. There was no evidence for changes in expression of glycolytic genes other than an apparent 2-fold decrease of glucose transporter *SLC2A3*. Intriguingly though, within the top-ranked 22 probe sets (Table S1), transcripts from eight Y-chromosome genes were down-regulated in the knockout line. On comparing the karyotype of HCT116 C3 with the WT line, we noted that the latter contains a subpopulation of cells retaining a Y chromosome while the HCT116 C3 line (and the HCT116 1C10 line) comprises only a single karyotype lacking the Y chromosome (Table S2). Thus the down-regulation of Y-chromosome transcripts may be an artifact resulting from clonal selection of a Y-minus variant.

Knockout of *ADPGK* in H460 (clone IIE5) caused a visibly greater alteration in gene expression (Figure 4B). Most notably the probe set for cadherin 11 (*CDH11*) was significantly decreased, by 150-fold, in H460 IIE5 (p = 0.03). No other differentially expressed transcripts were identified when the false discovery rate was controlled to 5% by the Benjamini-Hochberg method, a conservative statistical threshold that is appropriate when so few replicates are available. Nevertheless, plotting the expression differences of the top-ranked 50 probe sets in a heatmap shows a visible but not statistically significant distinction between WT H460 line and the IIE5 H460 knockout clone duplicate cultures (Figure 4C). While there was no evidence for changes in the abundance of glycolytic mRNAs, two mRNAs encoding regulators of cellular metabolism did appear to be differentially expressed just below the level of statistical significance (*PPARGC1A*, 3-fold downregulated; and *AKT3*, 2.7-fold upregulated). Cadherins 2, 6 and 10 were also differentially expressed >2.5-fold but below the level of significance. The top-ranked 150 probe sets from H460 (Table S3) were chosen for further analysis with the gene ontology tool DAVID ([27]; http://david.abcc.ncifcrf.gov/tools.jsp). Close to 50% of the input genes were associated with the plasma membrane and predicted to be glycosylated, 20% were localised to the extracellular space and ∼16% were associated with cell adhesion (Table S4). Analysis using the GeneSetDB pathway analysis tool [28] also revealed that the top-ranked 150 probe sets from H460 cells were significantly enriched (p≤0.05) for Gene Ontology (GO, http://www.geneontology.org/) categories of ‘Adherens junction organisation’ and ‘Cell junction assembly’, and for the Reactome (http://www.reactome.org/) molecular pathway categories of ‘Adherens junctions interactions’ and ‘Cell junction organisation’.

In order to confirm suppression of mRNA for cadherin 11 and other cell adhesion molecules that appeared to be differentially expressed following *ADPGK* knockout (*NCAM2*, *L1CAM* and *CDH2)*, their expression was quantified by qPCR in H460 WT and both the *ADPGK*-null clones IIE5 and IID10 (Figure 4D). *ADPGK* mRNA was also quantified by qPCR, and demonstrated low transcript abundance in the mutant clones consistent with nonsense-mediated decay. Expression of *CDH11* was strongly suppressed in the *ADPGK* knockouts (>10,000-fold in H460 IIE5 and ∼200-fold in H460 IID10). *NCAM2* and *L1CAM* RNA were decreased in the range of 30 to 40-fold and 3 to 4-fold, respectively, in both the *ADPGK*-null clones. *CDH2* was only decreased in H460 IIE5 (∼1000-fold). In contrast, this suppression of *CDH11, NCAM2 and LICAM* was not detected when *ADPGK* was knocked down to ∼ 80% of WT levels by siRNA (Figure S4) suggesting that complete loss of gene function may be required for this phenotype.

### Effect of *ADPGK* Knockout on Cell Survival and Glycolysis in HK2-depleted Cells and Under Anoxia and Hypoxia

**Figure 5:**
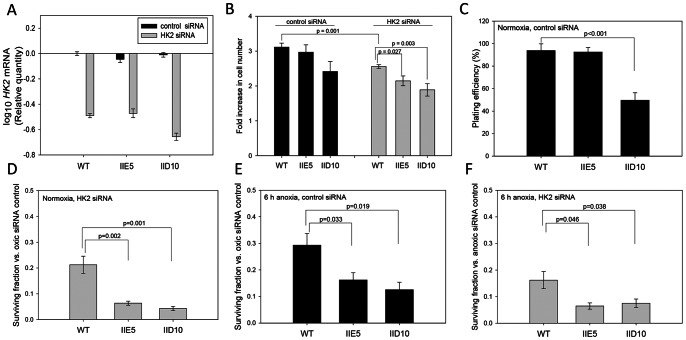
Knockout of *ADPGK* in H460 lowers clonogenic survival under anoxia and when *HK2* is knocked down. *HK2* was knocked down by siRNA in two independent experiments, combined here, each of which included three biological replicates for WT and two for each knockout clone. (A) HK2 mRNA by qPCR one day after siRNA transfection. (B). Cell number two days after siRNA transfection, at which time cells were replated for clonogenic assay. (C) Plating efficiencies of two days after transfection with control siRNA. (D) Effect of *HK2* siRNA on clonogenic surviving fraction two days after transfection, relative to cells transfected with control siRNA. (E) Effect of 6 h anoxia on clonogenic surviving fraction, relative to oxic controls, determined by plating cells in an anoxic chamber two days after siRNA transfection and transferring to an aerobic incubator 6 h later. (F) Effect of *HK2* siRNA on clonogenic surviving fraction after exposure to 6 h anoxia, relative to an equivalent anoxic exposure after control siRNA.

The above studies indicated little if any effect of *ADPGK* knockout on cell proliferation or plating efficiency (clonogenicity) under normal growth conditions. We therefore tested whether ADPGK might contribute to cell survival when glucose phosphorylation is restricted by treatment with *HK2* siRNA, or when glycolytic demand is increased under strictly anaerobic conditions (6-h anoxia). One day after transfection of H460 WT, IIE5 and IID10 with *HK2* siRNA or GC-matched control siRNA, *HK2* knockdown as measured by qPCR was in the range 70–80% (Figure 5A). Two days after transfection with control siRNA, cell numbers of knockout clones were not significantly different from WT, although a small trend towards reduced proliferation was noted for H460 IID10 as above (Figure 5B). *HK2* siRNA significantly reduced cell numbers in WT cultures relative to control siRNA, and both knockout clones showed a small but significant further decrease in cell number compared to *HK2* siRNA treated WT (Figure 5B). Plating efficiency of the cells treated with control siRNA, scored 10 days after re-plating cells, was also reduced for the IID10 line (∼50% of that for H460 WT), but not for IIE5 (Figure 5C).

The ability of glycolytic restriction (*HK2* siRNA) or increased glycolytic demand (anoxia) to further suppress clonogenicity was evaluated by calculating the surviving fraction (plating efficiency as a fraction of that for the normoxic control siRNA-treated cells). *HK2* siRNA killed 79% of H460 WT (surviving fraction 0.21 in Figure 5D) and the resulting colonies were smaller (40% reduction in mean colony radius; data not shown). Notably, both *HK2* siRNA transfected *ADPGK*-null cell lines showed an additional highly significant ∼4-fold loss of clonogenicity (Figure 5D), with no further change in colony size, compared to WT also treated with *HK2* siRNA. When cells were re-plated in an anaerobic chamber and exposed to 6 h anoxia before being transferred to an aerobic CO_2_ incubator, clonogenic survival of WT cells decreased by 71% (Figure 5E) and colony radius by 30% (not shown). Both H460 *ADPGK*-null cell lines were significantly more sensitive to killing under anoxia (Figure 5E), with no further change in colony radius. *HK2* knockdown in combination with anoxia had a similar effect on viability to that seen under aerobic conditions, again with a significantly greater effect in the *ADPGK*-null than WT lines (Figure 5F).

Increased sensitivity of both ADPGK knockout H460 clones to anoxia was confirmed in a second set of experiments, both with and without the glycolytic inhibitor 2-deoxy-D-glucose (Figure S5). These experiments clearly show that *ADPGK* has a protective effect against cell death under anoxia and when *HK2* is knocked down under aerobic or anoxic conditions in the H460 cell line.

A similar study to that for H460 cell lines was conducted for HCT116 WT and its *ADPGK*-null derivatives C3 and IC10 (Figure S6). In this case, the only statistically significant effect of *ADPGK* knockout was a modest decrease in plating efficiency (of the control siRNA-treated cells) which appeared to be more pronounced than in the experiment without control RNA transfection reported in Table 1. Knockdown of *HK2*, and exposure to anoxia for 6 h, caused less cell killing of HCT116 WT than H460 WT cells by a factor of ∼2, and knockout of *ADPGK* did not further increase killing by *HK2* siRNA or anoxia in the HCT116 background.

We further investigated whether prolonged exposure of hypoxia (0.2% oxygen in gas phase) has a similar effect on H460 *ADPGK*-null clones as shown for anoxia. All three cell lines proliferated at similar rates under hypoxia for 3 or 6 d, with no significant difference in the fold increase in clonogens per culture (Figure S7A). Similarly, *ADPGK* knockout had no consistent effect on growth of HCT116 clonogens under 0.2% oxygen for 3, 6, or 9 days (Figure S7B).

The apparent difference in effects of *ADPGK* knockout on survival of H460 cells under anoxia versus 0.2% oxygen led us to test activation of the unfolded protein response (UPR), which is more pronounced under severe than moderate hypoxia [29], [30]. *XBP1* splicing by the IRE-1 endonuclease, a marker of UPR [31], showed similar increases in H460 *ADPGK*-null clones and WT cells after 6-h anoxia (Figure S8).

### Effects of *ADPGK* Knockout on Glycolysis and Mitochondrial Metabolism

**Figure 6:**
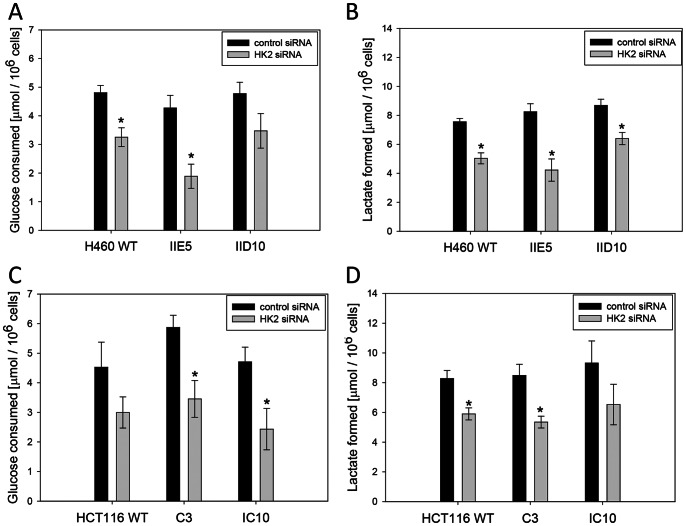
Knockdown of *HK2* suppresses anaerobic glycolysis in H460 and HCT116, but knockout of *ADPGK* has no effect. Glucose consumption (A, D) and lactate formation (B, E) were measured 2 days after transfection with control siRNA or *HK2* siRNA. Cells were re-seeded in fresh medium at 2×10^5^ per ml in 96-well plates in an anaerobic chamber, and the culture medium was assayed 6 h later. Values are mean and SEM for 3 independent experiments. Asterisks indicate a significant difference for HK2 siRNA relative to control siRNA (p<0.05).

**Figure 7:**
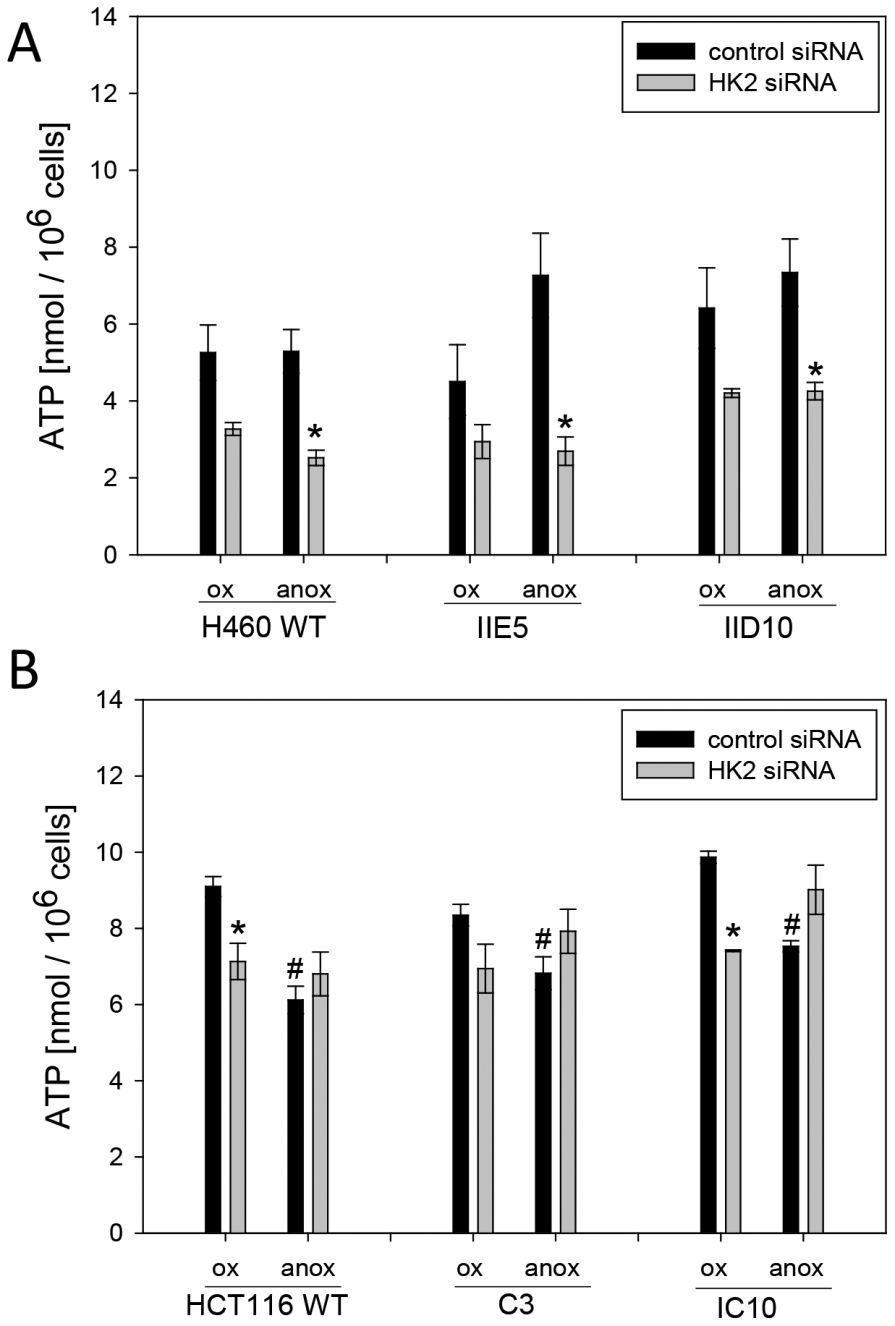
Steady state ATP levels are more sensitive to *HK2* suppression in H460 than HCT116 cells, but knockout of *ADPGK* has no effect in either. ATP was measured 2 days after transfection with control siRNA or *HK2* siRNA, following re-seeding in fresh medium for 6 h under normoxia (ox) or anoxia (anox). Values are mean and SEM for 3 biological replicates. Asterisks indicate significance (p<0.05) of *HK2* siRNA treated vs. siRNA control treated cells. Hash indicates p<0.05 for difference between anoxic vs. normoxic conditions.

We next tested whether the effects of *ADPGK* knockout on H460 survival under anoxia reflect a role of ADPGK in glycolysis. Glucose consumption and lactate formation during 6-h anoxia were suppressed when *HK2* was knocked down in H460 (Figure 6A, B) and HCT116 (Figure 6C, D) cell lines, establishing that glucose phosphorylation is rate-limiting for glycolysis under these conditions. Surprisingly, there was no evidence for a lower glycolytic rate in the *ADPGK* knockouts, and glycolysis in the knockouts was no more sensitive to *HK2* suppression. Similarly, knockdown of *HK2* suppressed steady-state ATP levels (under either aerobic or anaerobic conditions) in H460 cell lines, but this effect was no greater for any of the *ADPGK*-null clones (Figure 7A). Anoxia and *HK2* siRNA had only minor effects on ATP levels in HCT116 WT cells, consistent with the lesser effect of these treatments on clonogenic survival than in H460 cells, with no obvious differences in the *ADPGK* knockout clones (Figure 7B).

**Figure 8:**
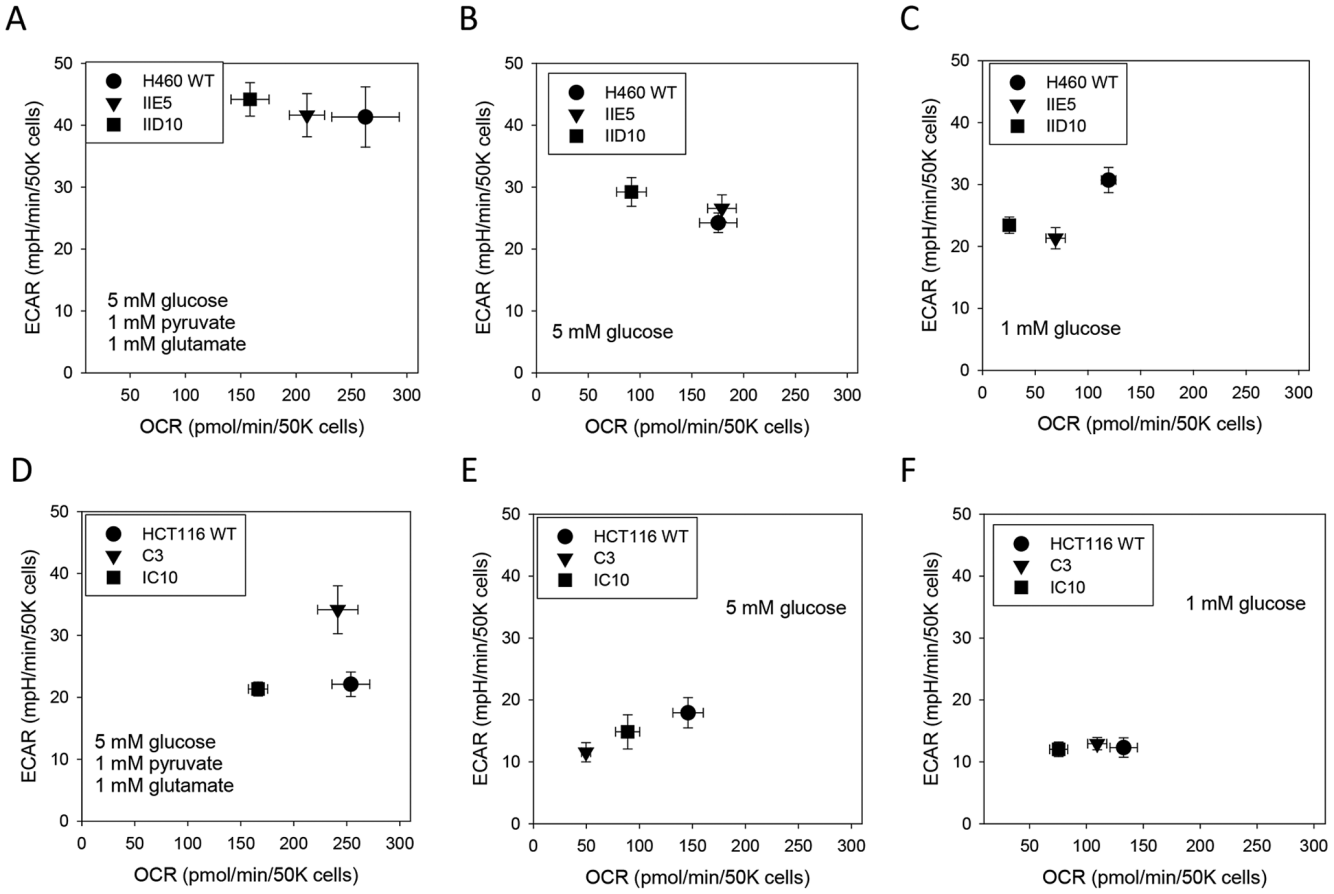
Reduced oxygen consumption rate (OCR) but not extracellular acidification rate (ECAR) in *ADPGK* knockout cells under aerobic conditions. Basal OCR and ECAR were measured in H460 (A–C) and HCT116 (D–E) *ADPGK* knockout cell lines and their respective wild types (WT) using a Seahorse XF analyser with monolayers in different media as indicated. Values are mean and SEM from two independent experiments with five biological replicates each.

To further investigate the potential role of ADPGK in glycolysis, we also evaluated rates of aerobic glycolysis using a Seahorse XF24 Extracellular Flux Analyser. WT and *ADPGK*-null cells of both genetic backgrounds were assayed in unbuffered DMEM media, and extracellular acidification rates (ECAR) and oxygen consumption rates (OCR) were measured simultaneously. Knockout of *ADPGK* had no effect on ECAR, a proxy measure of glycolytic flux [32], in H460 when assayed in media containing 5 mM glucose, 1 mM pyruvate and 1 mM glutamate (Figure 8A) or media containing 5 mM glucose exclusively (Figure 8B). However, when glucose was decreased to 1 mM both *ADPGK*-null cell lines showed a small but statistically significant (p = 0.002) reduction in ECAR compared to H460 WT (Figure 8C). In HCT116 cells, knockout of *ADPGK* had no effect on ECAR (Figure 8D-F), with the exception of clone HCT116 C3, which surprisingly showed an increase in ECAR in media containing pyruvate and glutamate additionally to glucose (Figure 8D). However, removal of pyruvate and glutamate from the media reduced ECAR in clone HCT116 C3 below that of HCT116WT cells (Figure 8E) and could suggest more complex interactions between multiple metabolic pathways in this particular clone.

Interestingly, knockout of *ADPGK* in both HCT116 and H460 cells resulted in clone- and glucose-dependent reductions in OCR. In H460 cells, the IID10 clone showed the most pronounced reductions in OCR, at all glucose concentrations, as did the IC10 clone in HCT116 cells. Residual oxygen consumption in the presence of the ATP synthase inhibitor oligomycin and complex III inhibitor antimycin A (i.e. non-mitochondrial oxygen consumption) was in most cases unchanged for the knockout lines relative to the parental cells (Figure S9).

**Figure 9:**
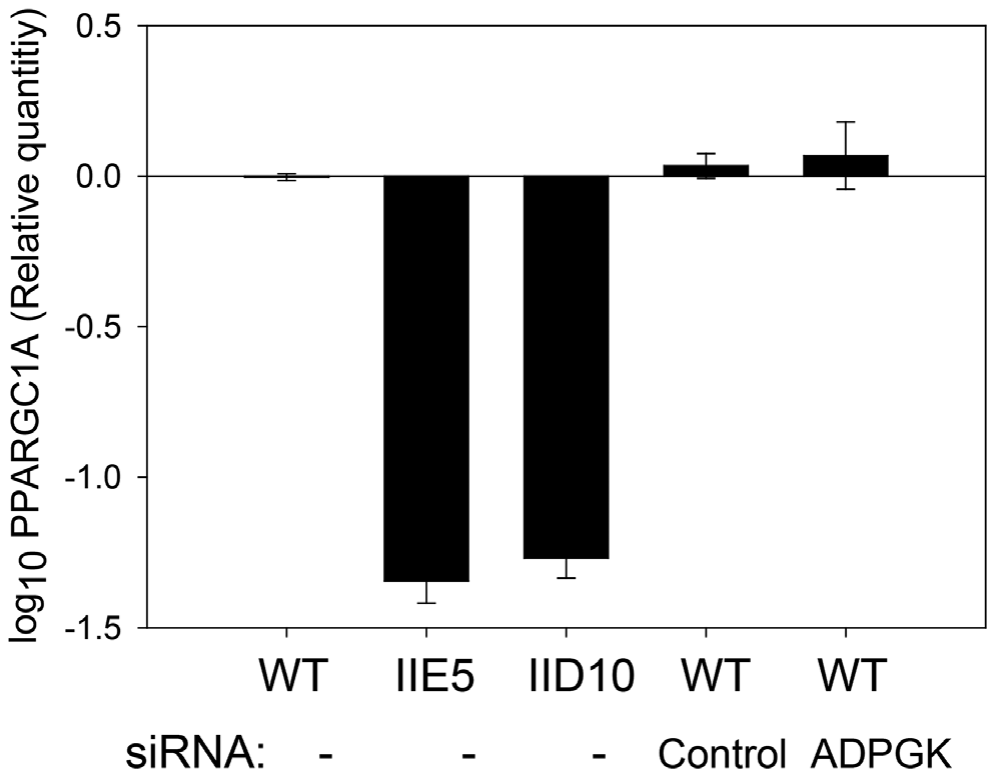
Effect of knockout of *ADPGK,* and its knockdown by siRNA, on expression of *PPARGC1A* in H460 cells. *PPARGC1A* expression was measured by qPCR in WT and the two *ADPGK-*null clones, and in H460 WT cells two days after treatment with control or *ADPGK* siRNA. Standard errors are shown for 3 replicate cultures with 3 technical replicates each.

To investigate whether these reductions in basal OCR were dependent on glycolytic flux, we examined OCR in media containing 5 mM glucose, 1 mM pyruvate and 1 mM glutamate, following the addition of 2-deoxy-D-glucose to block glycolytic flux. After the addition of 2-deoxy-D-glucose, ECAR decreased by ∼50%, while OCR was not altered, in all cell lines (Figure S10). These data suggest that impaired glucose oxidation does not account for the oxidative phenotype observed. The 3-fold reduction in expression of peroxisome proliferator-activated receptor gamma coactivator 1-alpha (*PPARGC1A*), a key regulator of mitochondrial biogenesis, in H460 clone IIE5 by microarray (Table S2) suggested a possible mechanism for the reduced OCR. Suppression of *PPARGC1A* in IIE5, and in the second H460 *ADPGK*-null clone IID10 was confirmed by qPCR which showed a 20-fold reduction in both clones compared to WT, although siRNA knockdown of *ADPGK* (which lowered *ADPGK* mRNA to 31±11% of control values in this experiment) did not suppress *PPARGC1A* (Figure 9). To further investigate potential consequences of reduced *PPARGC1A* expression, we measured mitochondrial mass, using Mitotracker staining (Figure S11A), and found no differences between any of the cell lines. Furthermore, we found no differences in electron transport chain complex protein expression in HCT116 cells (Figure S11B). However, in H460 cells, protein expression of components of the electron transport chain complexes I and II appeared to be reduced in the IID10 clone and was associated with a loss in mitochondrial membrane potential (Figure S11C). These data suggest that ADPGK can induce clonal dependent alterations in mitochondrial metabolism.

### *ADPGK* Knockout Tumours: Growth and Hypoxic Fraction of Xenografts

**Figure 10:**
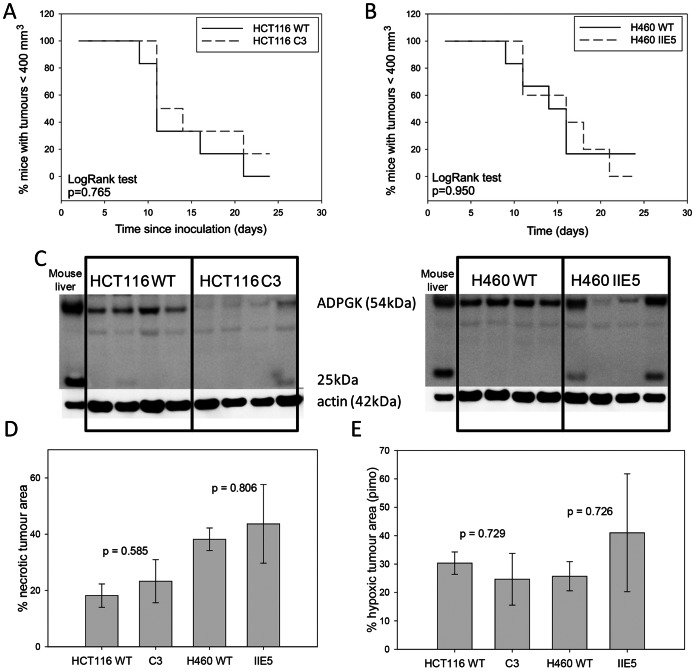
Tumour xenograft growth from *ADPGK* WT and knockout cell lines. Kaplan-Meier plots showing time for HCT116 (A) and H460 (B) tumours to reach endpoint. 5-6 mice/group. (C) Immunoblots of lysates from fresh frozen tumours and mouse liver at the tumour growth endpoint. (D) Proportion of tumour area which is necrotic, as determined by H&E staining of histological sections at endpoint (4 tumours/group). (E) Proportion of tumour sections positive for the hypoxia probe pimonidazole, by immunostaining, in the same tumours as for D.

To investigate whether *ADPGK* influences xenograft growth and/or hypoxic fraction in tumours, xenografts were grown from two *ADPGK* knockouts, HCT116 C3 and H460 IIE5, and their WT counterparts. No difference in growth as subcutaneous tumours was found for the *ADPGK* knockout clones (Figure 10A/B). Western blots from fresh frozen samples of each tumour that reached the endpoint size showed a reduction of the 54-kDa ADPGK band in most of the ADPGK knockout tumours, but a residual band of varying intensity was evident (Figure 10C). This presumably reflects a variable murine stromal component, given that the antibody also recognised murine ADPGK in mouse liver samples; this is consistent with the presence of a cross-reactive 25 kDa band in mouse liver and in the *ADPGK* knockout tumour samples with the most intense 54 kDa bands (Figure 10C).

H&E staining and immunohistochemistry for the hypoxic probe pimonidazole did not reveal any differences in necrotic or hypoxic tumour area for any of the groups or cell lines (Figure 10D and E). The analysis was not biased by the tumour size, since there was no significant difference in tumour weights between groups (data not shown). In a second small study with HCT116 WT and C3 xenografts, no obvious difference was seen in staining for pimonidazole, the proliferation marker bromodeoxyuridine or the TUNEL apoptosis assay (Figure S12).

## Discussion

In this study we use a pair of ZFNs, custom-designed to target a genomically unique sequence in the *ADPGK* gene (Figure 1A), to generate human tumour cell lines with frameshift mutations at the target site and which completely lack ADPGK protein by immunoblotting. The truncated proteins predicted from sequencing the region near the ZFN target site represent 62–89 amino acid N-terminal fragments which would not be detected by the antibody we used, which recognises a C-terminal epitope [13]. However, these truncated proteins would almost entirely lack the ADP kinase catalytic domain (amino acids 52–497) and particularly the likely active site aspartate at position 481 and metal ion binding residues at 297 and 328 (www.uniprot.org), and are thus expected to be catalytically inactive. In addition the low abundance of the mutant ADPGK transcripts (Figure 4D), consistent with nonsense-mediated decay, indicates that any such N-terminal fragments are likely to be poorly expressed.

It is of note that two of the four ADPGK-null clones we isolated harboured homozygous mutations, including identical mutations in all three alleles in H460 IIE5. It is presumed that reduction to homozygosity occurred in these cases through ongoing ZFN-induced cutting of the wild type allele, resulting in homology-directed repair with the mutant allele as template, as has been suggested previously [33], [34]. The frequency with which multi-allelic knockout was achieved was ∼ 1.5%, with 2/143 screened HCT116 clones lacking *ADPGK* expression. This contrasts to the much higher proportion of mutations at the ZFN target site as demonstrated by the Surveyor mutation detection assay (Figure 1B), which detects monoallelic mutations as heteroduplexes (predominantly with the WT sequence). This suggests that reduction to homozygosity is a relatively infrequent event in the HCT116 cell line, leading to a low recovery of ADPGK-null clones. We have not been able to rigorously exclude the possibility that ADPGK-null clones are selected against because the genotype compromises cell proliferation or survival. The loss of mutations from ZFN-transfected pools over time (Figure S1A) would be consistent with this interpretation, except that most of the mutations in these pools are almost certainly monoallelic; the lack of effect of *ADPGK* knockdown by RNAi on viability and proliferation of HCT116 cells [13] indicates that haploinsufficiency does not result in a lethal phenotype. The observation of a similar progressive loss of *POR* mutations when HCT116 cells are transfected with a *POR* ZFN pair (Figure S1B), despite apparently more efficient recovery of null mutants (3/14 clones), suggests that the declining mutant frequency reflects compromised proliferation of the cells with highest plasmid copy number, possibly reflecting toxicity of highly expressed ZFNs [35]–[37]. Nonetheless, the low yield of ADPGK-null clones leaves open the possibility that secondary mutations or epigenetic changes could have rescued the rare *ADPGK* knockouts we isolated.

The efficiency of generation of ADPGK-null clones seemed to be even lower in H460, which required two rounds of ZFN transfection with 0/151 null lines in the first round and 5/121 on re-transfection of candidate partial knockouts; this lower efficiency may in part reflect the presence of three *ADPGK* alleles in this line. As yet there is little information to provide comparison with the efficiency of multi-allelic gene disruption by ZNF in other mammalian cell lines. Much higher rates have been reported for genes that are X-linked [38] or otherwise functionally haploid [39], or when the knockout provides a selectable phenotype [40], [41], but rates vary widely at other loci [33], [39], [40], [42]. The difficulties in distinguishing between low efficiency of multi-allelic knockout and rescue by compensating genetic/epigenetic changes with ZFN technology is likely to be particularly problematic with tumour cells given the dual problems of genomic instability and high gene copy number at many loci.

Clonal heterogeneity in tumour cell lines also represents a challenge for use of ZFN in that knockout clones and parental lines are unlikely to be strictly isogenic, and phenotypic differences may thus not have a causal relationship to the targeted mutations. Heterogeneity in the HCT116 line was evident in the karyotypic analysis; the parental line comprised subpopulations with and without a Y-chromosome as well as a polyploid variant, which is consistent with the karyotype reported by ATCC, while the *ADPGK* knockout clones were derived from the Y-minus pseudodiploid variant (Table S2) and lacked Y-chromosome transcripts (Table S1). Ideally a larger number of clones would have been evaluated in the present study, but this was precluded by the very low efficiency with which multi-allelic knockouts were recovered. Clonal heterogeneity is likely to be an important confounding factor in ZFN gene editing studies with tumour cell lines, and suggests that it would be advantageous to use cell lines that are relatively competent for homology-directed repair which would be expected to minimise genotypic diversity in the parental line as well as maximising gene conversion at the target locus (potentially leading to higher efficiency recovery of homozygous mutations).

Despite the low efficiency in production of null clones using ZFN, knockout of *ADPGK* in H460 resulted in a marked phenotype in relation to clonogenic survival under anoxia or when glycolysis is restricted with siRNA against *HK2* (Figure 5D-F). This phenotype was consistent in both H460 clones, but appears to be cell line dependent given that no such effects were observed in the two HCT116 knockout clones. This difference might reflect the higher ADPGK protein levels in H460 than HCT116 [13], or differences in ADPGK activity as a result of post-translational modification. The mechanism of transient activation of ADPGK during T-cell receptor signalling is unknown, but it occurs in the absence of a change in protein levels and is dependent on PKC-**θ** [18] suggesting a possible role for phosphorylation. Bioinformatic analysis using NETPHOS 2.0 [43] with a threshold of 0.5 suggests that 18 serine and 3 threonine residues of full-length human ADPGK are potential phosphorylation sites, but this has yet to be investigated experimentally. In relation to the cell line dependence of ADPGK knockout, it is perhaps also relevant that HCT116 cells were more resistant to these glycolytic stresses as indicated by a lesser effect of HK2 suppression and anoxia on cell survival (Figure S6) than for H460. In our previous siRNA study [13], knocking down *ADPGK* by 70–90% in H460 cells did not increase sensitivity to clonogenic cell killing under anoxia. Thus ZFN-mediated knockout may provide a clearer phenotype than RNAi, as also suggested in the present study by the strong reduction in *PPARGC1A* expression on knockout of *ADPGK* but not when it was knocked down with siRNA (Figure 9).

Given the clear effect of knocking out *ADPGK* on clonogenic survival of H460 under these stress conditions (Figure 5), we were surprised to find no observable effect on glycolysis in either H460 clone (or in the HCT116 knockouts). These studies used two independent measures of glycolysis (glucose consumption/lactate production in bulk cultures, and extracellular acidification rate in unbuffered media with a Seahorse analyser), and failed to reveal any defect in glycolysis even under conditions of *HK2* knockdown and anoxia (Figure 6). In addition, knockout of *ADPGK* also did not further compromise steady-state ATP levels in cells in which glycolysis was inhibited with *HK2* siRNA (Figure 7). Thus the effect of *ADPGK* on survival of H460 cells under these stress conditions appears not to be mediated by a direct effect on glycolytic flux. The studies with the Seahorse analyser also evaluated mitochondrial oxygen consumption in aerobic cultures, and interestingly, demonstrated a reduction in most *ADPGK* knockout clones in most media (Figure 8). This decrease in respiration on knockout of *ADPGK* is the reverse of the reported suppression of respiration in T-cells when ADPGK activity is stimulated through T-cell receptor/diacylglycerol signaling [18]. In addition, there was no consistent evidence for changes in oxygen consumption in the presence of oligomycin and antimycin A in the knockouts (Figure S9), as might be expected if ADPGK increases generation of ROS as in activated T-cells [18]. Thus *ADPGK* does not appear to participate in an analogous signalling pathway in HCT116 or H460 tumour cells under the conditions we have tested. The marked decrease in expression of *PPARGC1A* in the H460 knockouts (Figure 9) suggested that compromised mitochondrial biogenesis might be responsible for the decrease in oxygen consumption. In support of this hypothesis, we did observe a decrease in electron transport chain complex I and II expression, which could limit the reduction of the electron transport chain and ultimately reduce oxygen consumption. Consistent with this finding, we also observed a decrease in mitochondrial membrane potential in the IID10 H460 clone. It is unclear whether this is due to a direct effect of ADPGK on mitochondrial biogenesis processes, or due to feedback to these same processes from altered metabolic flux. However, in an effort to explain the difference between our findings and those observed with oxygen consumption in activated T-cells, it is possible that these effects could also be context-dependent and determined by specific signaling to ADPGK, as we have suggested for the wider functions of ADPGK. Furthermore, variability between clones leaves open the question as to whether changes in oxygen consumption represent a real effect of *ADPGK* or a manifestation of clonal heterogeneity.

Given the important role of hypoxia as a driver of tumour angiogenesis [44], we sought to determine whether the reduced survival of H460 *ADPGK* knockouts under anoxia *in vitro* translates into compromised growth as tumour xenografts. *ADPGK* knockout had no discernible effect on growth of the H460 or HCT116 clones, and steady state hypoxic fractions were unaffected in the resulting tumours (Figure 10). This apparent difference between *in vitro* and *in vivo* phenotypes let us to evaluate effects of moderate hypoxia (0.2% O_2_ gas phase) as distinct from anoxia *in vitro*; even for the H460 line showing greater sensitivity to anoxia, proliferation of clonogens under hypoxia was not compromised by knockout of *ADPGK* (Figure S7). Thus moderate levels of hypoxia in tumours, sufficient to induce HIF-1 stabilisation and angiogenesis, do not appear to result in *ADPGK* dependence. Our results suggest that *ADPGK* may not be a useful target for cancer therapy, although it needs to be born in mind that the current investigation explores just two clones from each of two human tumour cell lines. The intriguing observation of a clear effect of *ADPGK* on survival of H460 cells under certain stress conditions *in vitro* indicates that a deeper understanding of its role in tumour cell biology is needed. In particular, the effect on survival but not apparently glycolysis (despite the demonstrated ability of purified ADPGK to catalyse ADP-dependent glucose-6-phosphate formation [12], [13]) raises the question whether this ancient gene might have been co-opted for other functions during metazoan evolution, perhaps analogous to the roles of mitochondrially-bound HK2 in suppressing apoptosis and ROS generation [45], [46] and of PK-M2 as a co-activator of HIF-1 [47]. The recent demonstration of the diversion of glycolytic flux by ADPGK to hyper-reduce ubiquinone and drive mitochondrial generation of ROS, in response to T-cell receptor signaling, also raises the possibility that ADPGK might contribute to elevated ROS production in some tumour cells. However, we observed no consistent difference in oxygen consumption in the cells lines examined in the present study following antimycin exposure, which in part reflects mitochondrial ROS generation, nor consistent alterations in mitochondrial membrane potential. This suggests that modulation of ADPGK levels in H460 and HCT116 cells did not affect the reduced state of the respiratory chain or ROS production. The gene expression changes observed in H460 cells suggest that ADPGK might also regulate expression of cell adhesion molecules in some contexts, indicating a possible avenue for further investigation. In addition, *Adpgk* knockout mouse embryonic stem cells are now available, providing another important opportunity for evaluation the role of this enigmatic gene in mammalian biology.

## Materials and Methods

### Ethics Statement

Animal experiments were conducted under protocols approved by the University of Auckland Animal Ethics Committee (approval number CR830).

### Cell Culture and Generation of *ADPGK*-null Lines using Zinc-finger Nucleases

H460 and HCT116 cell lines were obtained from the American Type Culture Collection (ATCC), VA and used within 90 days passage from frozen stocks confirmed to be mycoplasma negative using a PCR-Elisa kit (Roche, Switzerland). All cell lines were grown in alphaMEM (Invitrogen, CA), supplemented with 5% foetal bovine serum (FBS). Cells were subjected to anoxia (0% oxygen in a 5% CO_2_/5% H_2_/90% N_2_ gas phase) in a Bactron Pd calatyst anaerobic chamber (Sheldon Manufacturing, OR), using plasticware and solutions equilibrated to anoxia for at least three days before the experiment.

Plasmids for expression of a pair of custom-designed CompoZr™ Fok1 ZFNs against two 18 base pair target sites in the first exon of the human *ADPGK* gene (chromosome 15∶73,043,636-73,076,126) were purchased from Sigma Aldrich, MO. The targeted region is: 5′-CCGGGACCCGTCTCCCCCgagggCCGGTTGGCGGCAGCCTGG-3′, where the lower case bases indicate the untargeted Fok1 nuclease site. A second ZFN pair, also purchased from Sigma Aldrich, targets the sequence CATAGATGCGGCCAAGGTgtacatGGGGGAGATGGGCCG in the 8^th^ exon of the *POR* gene. Plasmids were transfected into cells using Lipofectamine™ LTX and Plus reagent (Invitrogen, CA) according to the manufacturer’s instructions (for generation of HCT116 C3), or alternatively by nucleofection (Lonza, Switzerland) using Nucleofector™ solution V and programs D-032 for HCT116 and T-030 for H460. Co-transfection with a GFP-coding plasmid (pEGFP-N1, Clontech) and subsequent fluorescence-activated cell sorting (Becton Dickinson FACSVantage Cell Sorter) was used in some cases to enrich for positively transfected cells (for generation of clones HCT116 C3, H460 ID10 and IIE5). ZFN-transfected cells were cloned by limiting dilution in 96 well plates, and after growing for 7 days wells containing a single colony were trypsinised and expanded in duplicate wells, one of which was trypsinised and extracted (Laemmli buffer, Bio-Rad Laboratories, CA) for screening by western immunoblotting. Clones H460 IID10 and HCT116 IC10 were found to be contaminated with mycoplasma; all data presented below (except Figures 3, 5, 6 and S6) was obtained after elimination with Plasmocure™ (Invivogen, CA).

### Surveyor Mutation Detection Assay

Genomic DNA was extracted according to the manufacturer’s instructions using the GenElute™ Mammalian Genomic DNA Miniprep Kit (Sigma-Aldrich, MO). 100 ng of DNA, primers 5′-GTAGCGCTTGTGTCGACATCGGCGC-3′ and 5′-CAGACGTCCGACACTTCTCGCCAAG-3′ and Extract-N-Amp™ Blood PCR Ready Mix™ (Sigma-Aldrich, MO) were combined to amplify the *ADPGK* ZFN cutting site. The primers for the *POR* ZFN site were 5′-CAAAAGGGCTCATTTCCTTAAAATC-3′ and 5′-GCTAAGTGAGCTCAGTACAAACTGG-3′. The Surveyor mutation detection assay, using the CEL-II nuclease, was performed as per the manufacturer’s instructions (Transgenomic Inc., NE). Products were resolved on a 2% agarose gel and band densitometry was undertaken using Image J software.

### Sequencing of the ZFN Cutting Site

Genomic DNA from WT cells and knockout clones was amplified by PCR using primers surrounding the ZFN cut site (ADPGK_P1): 5′-GGGGACAAGTTTGTACAAAAAAGCAGGCACCGTAGCGCTTGTGTCGACATCGGCGC-3′ and 5′-GGGGACCACTTTGTACAAGAAAGCTGGGTTCCAGACGTCCGACACTTCTCGCCAAG-3′, which include attB sites for Gateway cloning (underlined). The products were cloned into pDONR221 via Gateway™ cloning (Invitrogen, CA) and the plasmid was transformed into *E. coli*. Eight colonies for each cell line were picked for plasmid extraction and DNA sequencing. To uncover large deletions, a second primer set (ADPGK_P2) further away from the ZFN cut site was employed: 5′-ACAGCAGGTCCCATAGCAAC-3′ and 5′-CCCACCAATTCATATGCTGA-3′.

### Western Immunoblotting

SDS-PAGE and western blotting was performed as previously [13]. Primary antibodies were mouse monoclonals against ADPGK and HK2 (Abnova, Taiwan), rabbit polyclonal anti-GAPDH (Abcam, UK) and mouse monoclonal anti-actin (Chemicon International). Secondary HRP-coupled anti-mouse and anti-rabbit antibodies were from Santa Cruz Biotechnology, CA.

### Karyotyping and qPCR Copy Number Variation (CNV) Assay

Log-phase cultures were treated with colcemid (50 ng/ml, 1 h) and metaphases were prepared for Trypsin-Giemsa staining using standard methods [48], [49]; 20 metaphases were analysed for each cell line. qPCR was performed using the Platinum SYBR Green qPCR SuperMix-UDG with ROX module (Invitrogen, CA) and 10 ng of genomic DNA as template. Reactions were performed in quadruplicate in a total volume of 10 µl using the ABI 7900HT Fast Real-Time PCR System (Applied Biosystems, CA) with the following program: 50°C 2 min, 95°C 10 min, 40 cycles of 95°C 15 s, 60°C 30 s, 72°C 30 s. Primer sets were designed to assess copy number at three different sites, within exon 7 of *ADPGK* gene, immediately down and upstream of the ZFN cut site (see Figure 1A): CNV1 (5′-CTCACCCAACATCCTGGTCT-3′, 5′-TTTGTCCTTAGCGGCTCTGT-3′); CNV2 (5′-TTGCTAATCCCGCTTTGTCT-3′, 5′-ACGCGTCTTCAAGAAGTTCAG-3′); CNV3 (5′-AGGCCTGGAAGAGGACAAGT-3′, 5′-ATCCCCAAATAGAGGGATGG-3′). All values were normalized relative to the house keeping gene *RPP21*∶5′-AGGTTCTGGGTTGGTGTGAG-3′, 5′-TGTGTTTGGCAAGGGTTGTA-3′. For analysis, the software package SDS 2.3 and RQ manager from Applied Biosciences, CA was used. Copy numbers were calculated by using the formula 2·2^−ΔΔCt^ (Ct = cycle threshold). HCT116 with two *ADPGK* copies (www.sanger.ac.uk/cgi-bin/genetics/CGP/cghviewer/CghViewer.cgi) was employed as calibrator.

### Proliferation, Cell Volume, Cell Cycle Distribution and Clonogenicity

Cell number and cell volumes were determined from trypsinized monolayer cultures (combined with the original medium to include any floating cells) using a Beckman Coulter counter (Z2 Coulter Particle Count and Size Analyzer). Cell cycle distributions were determined after fixation in 70% ethanol and staining with propidium iodide (10 µg/ml in 0.1% Triton X-100, 100 µg/ml DNase-free RNase A in PBS) for 30 min using an LSR II flow cytometer (BD) and analyzed with FACSDIVA software (BD). 100 or 1000 cells were seeded in triplicate for clonogenic assay as previously [13].

### mRNA Microarrays

Cell lines were seeded at 2.5×10^4^ cells in T25 flasks. After four days, culture medium was removed and 750 µl TRIZOL® RNA extraction reagent (Invitrogen, CA) was added. RNA was extracted using Qiagen RNeasy Mini columns following the manufacturer’s instructions. The quality of the total RNA (1 µL) was checked using the Eukaryotic Total RNA 6000 Nano Assay on an Agilent 2100 Bioanalyser according to the manufacturer’s protocol (Agilent, CA). Subsequently, total RNA was processed using the Affymetrix Whole Transcript (WT) Sense Target Labeling Assay Rev. 5, and fragmented, labelled single-stranded DNA was hybridised to Affymetrix GeneChip® Human Gene 1.0 ST arrays at 45°C in a hybridisation oven for 17 h. The arrays were washed and stained using the Affymetrix protocol FS450 _0001 in an Affymetrix GeneChip Fluidics Station 450, and scanned in an Affymetrix GeneChip Scanner 7G. Data analysis was undertaken using R version 2.11.0 with the following Bioconductor packages and libraries: affy, limma, gdata, gplots and hugene11stprobeset.db. Specifically, raw microarray data at the probe level was normalised using the Robust Multiarray Analysis method with background correction (RMA [50], provided by the R ‘affy’ package). For quality control, Expression Console software (Affymetrix, CA) and R were used. The raw and normalized array data, and the relevant MIAME compliant metadata, can be accessed from GEO with accession number GSE39497 (www.ncbi.nlm.nih.gov/geo/query/acc.cgi?acc=GSE39497).

### Quantitative Real-time PCR (qPCR)

qPCR was performed as detailed in [13] with additional primer pairs for *CDH11* [51]; *CDH2* [52]; *NCAM2* [53]; *L1CAM* [54] and *PPARGC1A* [55].

### Measurement of Glycolysis, ATP and Oxygen Consumption

*HK2* siRNA treatment, glucose and lactate measurements and ATP assays were performed as previously [13]. Extracellular acidification rate (ECAR) and oxygen consumption rate (OCR) were measured with a Seahorse XF analyser (Seahorse Bioscience, MA) in unbuffered DMEM (no serum, Sigma-Aldrich, MO) containing either 5 mM glucose, 1 mM glucose or 5 mM glucose/1 mM pyruvate/1 mM Glutamax™ (Invitrogen, CA) as substrates. Plates were incubated for 60 min in a non-CO_2_ incubator at 37°C before three basal measurements were undertaken determining oxygen and proton concentration in the medium. Then the glycolytic inhibitor 2-deoxy-D-glucose (100 mM) was injected, or (in separate studies) the ATP synthase inhibitor oligomycin (1 µM) and the complex III inhibitor antimycin A (1 µM) were injected serially, with three further measurements after each addition. Seahorse plates had all media removed, without disrupting the cell monolayer, and were frozen at −80°C, immediately after the conclusion of the assay. Cell number in each well was determined using the CyQUANT kit (Invitrogen, CA), according to manufacturer’s instructions. All OCR and ECAR values were normalized to 5×10^4^ cells.

### Tumour Xenografts

Immunodeficient female NIH-III nude mice (NIH-Lyst^bg^Foxn1^nu^Btk^xid^), approximately 21 g body weight, were housed in groups of 4–6 under specific pathogen-free, controlled ambient conditions (22±2°C, 12 h day/night lighting cycle) with standard rodent diet (Harlan Teklad diet 2018i) and sterile water supplied *ad libitum*. Tumour cells (7×10^6^/site) were inoculated subcutaneously on the right flank. Tumours were measured every 2–3 days using digital calipers and volumes were calculated as width^2^×length×π/6. Tumours were harvested at approximately 1 cm^3^, 90 min after dosing the mice intraperitoneally with 1000 mg/kg bromodeoxyuridine (Sigma-Aldrich, MO) and/or 60 mg/kg pimonidazole (HPI Inc.). Mice were culled by cervical dislocation and tumours were excised immediately. Half of each tumour was frozen in liquid nitrogen, stored at −80°C and subsequently pulverized using a liquid N_2_-cooled Biopulveriser (BioSpec Products, OK) and lysed using Laemmli buffer. The other half was fixed with 10% neutral buffered formalin for 36–48 h, then 70% histology alcohol (18∶1:1 ethanol/methanol/isopropyl alcohol) before paraffin embedding and cutting 5 µm sections.

### Immunohistochemistry

FFPE sections were deparaffinised and antigen retrieval was carried out using 10 mM sodium citrate buffer pH 6.0 in a pressure cooker for 25 min, followed by cooling on ice for 10 min. Non-specific binding was blocked by incubation with 10% goat serum in TBS (0.2 M Tris, 1.37 M NaCl pH 7.6) for 1 h in a humidified chamber. FITC-pimonidazole monoclonal antibody (Hypoxyprobe™, HPI Inc.) was diluted 1∶25 in 5% goat serum in TBS and incubated overnight at 4°C in a dark humidified chamber. The next day, coverslips were mounted with ProLong® gold antifade reagent (Invitrogen, CA). Slides were stored at 4°C in the dark until imaging with a Nikon TE2000E Inverted microscope (montage function). The same tumour section were then stained with hematoxylin & eosin (H&E, Sigma-Aldrich, MO) and re-imaged. The percentage of necrotic and hypoxic regions in tumour sections was calculated using ImageJ.

### Statistics

Descriptive statistics report the mean and SEM for biological replicates (separate cultures) unless otherwise indicated. Significance of difference between two groups was tested using Student’s t-test, and significance between more than two groups was evaluated by one-way ANOVA/Holm Sidak.

## Supporting Information

Figure S1 Changes in mutation frequency in HCT116 pools during growth after transfection with ZFNs. HCT116 cells were co-transfected with a GFP plasmid and with plasmid pairs for *ADPGK* ZFNs (A) and *POR* ZFNs (B) by nucleofection, and 24 h later 25% of the cells with the brightest GFP fluorescence were sorted. Genomic DNA was prepared on the indicated day after transfection (day 1 sample obtained immediately after sorting), and the mutation frequency was assessed using the Surveyor mutation detection assay. For *ADPGK* the positive control is a reference DNA sample from *ADPGK* ZFN-treated K562 cells, provided by Sigma Aldrich, showing the predicted Surveyor products (256 and 217 bp, arrows). For *POR* the positive control is from a POR null clone (Hko3) with two mutant alleles (a M263L missense mutation and a one-bp insertion) at the ZFN target site that gives predicted Surveyor products of 417 bp and 248 bp (arrows).
(TIF)

Figure S2 Characterisation of the large *ADPGK* deletion in HCT116 clone C3. Yellow marks the binding site for primer pair ADPGK_P2. Grey marks the WT sequence deleted in HCT116 C3. The *ADPGK* start codon ATG is underlined and the ZFN recognition site is marked in red (with cutting site in lower case).
(TIF)

Figure S3 *ADPGK* copy number of HCT116, H460 and SiHa. Copy number was determined via qPCR with HCT116 as calibrator with known copy number of 2 (according to Sanger Institute, cancer genome project). Primers amplify a 175 bp sequence located within exon 7 of the *ADPGK* gene. Error bars show the SEM for four technical replicates. Boxed numbers indicate copy number identified by the Sanger Institute (www.sanger.ac.uk/cgi-bin/genetics/CGP/cghviewer/CghViewer.cgi). All cell lines were obtained from the American Type Culture Collection (ATCC), VA.
(TIF)

Figure S4 No decreased expression of cell adhesion molecules when *ADPGK* is knocked down in H460. RNA from three separate experiments was isolated one day after transfection (RNAiMAXTM, Invitrogen, CA) with both control siRNA and *ADPGK* siRNA (Invitrogen, CA). RNA was transcribed into cDNA and analysed by qPCR. All values are normalised against 18S rRNA, and error bars represent the standard error of three biological replicates each.
(TIF)

Figure S5 *ADPGK* KO reduces anoxic cell survival but not lactate formation in H460, and 2-deoxy-D-glucose (2DG) has no effect on these parameters. H460 cells (WT, KO clone IIE5 and IID10) were plated in an anoxic chamber for 2 h before being treated with 2 concentrations of 2DG (1 mM, 10 mM, Sigma-Aldrich, MO) or saline only for 4 h. Graphs show results from 3 independent experiments with 3 experimental replicates each. A. Anoxic surviving fraction measured by clonogenic assay after exposure to 6 h anoxia. B. Lactate formation measured in culture medium after 4 h exposure to saline or 2DG.
(TIF)

Figure S6 Knockout of *ADPGK* in HCT116 does not affect clonogenic survival under anoxia and when *HK2* is knocked down. *HK2* was knocked down by siRNA in two independent experiments, combined here, each of which included three biological replicates for WT and two for each KO clone. (A) *HK2* mRNA by qPCR one day after siRNA transfection. (B). Cell number two days after siRNA transfection, at which time cells were replated for clonogenic assay. (C) Plating efficiencies of two days after transfection with control siRNA. (D) Effect of *HK2* siRNA on clonogenic surviving fraction two days after transfection, relative to cells transfected with control siRNA. (**E**) Effect of 6 h anoxia on clonogenic surviving fraction, relative to oxic controls, determined by plating cells in an anoxic chamber two days after siRNA transfection and transferring to an aerobic incubator 6 h later. (**F**) Effect of *HK2* siRNA on clonogenic surviving fraction after exposure to 6 h anoxia, relative to an equivalent anoxic exposure after control siRNA.
(TIF)

Figure S7 Knockout of *ADPGK* in H460 (A) and HCT116 (B) does not affect cell growth or clonogenic survival under chronic hypoxia. Cells were seeded at 20,000, 1000 or 200 cells/well into 24-well plates and exposed to 3, 6 or 9 days of hypoxia (0.2% oxygen in gas phase), respectively. Cell number was measured using a Beckman Coulter counter and cells were re-plated for 10 days to measure clonogenic survival. Asterisks indicate significance (p<0.05) compared to WT. For HCT116 cell lines, two separate experiments were performed. Hypoxia (0.2% oxygen 5% CO_2_/N_2_) was achieved with an anaerobic glove box system and an oxygen controller (Coy Laboratory Products, Inc.). For long-term exposure, plates were partially enclosed in plastic bags containing trays of water to maximise humidity.
(TIF)

Figure S8 *XBP1* splicing under anoxia in H460 cell lines. RNA from H460 WT and two ADPGK-null clones was extracted after exposure to either 6-h normoxia (1 culture each) or anoxia (3 cultures each). RNA was transcribed into cDNA and analysed by qPCR. Two primer pairs, one reporting on total *XBP1* (GGCATCCTGGCTTGCCTCCA; GCCCCCTCAGCAGGTGTTCC) and one reporting on spliced *XBP1* (CGCTTGGGGATGGATGCCCTG; CCTGCACCTGCTGCGGACT) were used. All values were normalised against 18S rRNA, and the ratio between spliced and total *XBP1* was calculated. Error bars represent standard error.
(TIF)

Figure S9 Inhibition of oxygen consumption by oligomycin and antimycin. Oxygen consumption rate (OCR) in ADPGK-null clones and parental (WT) cell lines in the indicated culture media, determined using a Seahorse XF Analyser, and normalised to 50,000 cells. Values are mean and errors are SEM for five biological replicates, and are derived from two timepoints for basal conditions and three measurements after addition of oligomycin (1 µM) and antimycin A (1 µM). Asterisks indicate significance (p<0.05) of differences from the respective WT.
(TIF)

Figure S10 Inhibition of glycolysis by 2-deoxy-D-glucose (2DG). Five replicate cultures of each cell line were seeded in complete medium (5 mM glucose, 1 mM pyruvate, 1 mM glutamate), and EACR and OCR were measured with a Seahorse XF Analyser. After addition of 100 mM 2DG (Sigma-Aldrich) measurements were repeated. Asterisks indicate significance (p<0.05) of differences from the respective WT.
(TIF)

Figure S11 Knockout of *ADPGK* in H460 and HCT116 has no effect on mitochondrial mass (A), but has clone-dependent effects on electron transport chain subunit expression (B) and mitochondrial membrane potential (C). For (A), cells were seeded at 25,000 cells per well in black, clear bottom 96 well plates. 24 hours later, cells were stained with 50 nM Mitotracker Green FM (Life Technologies) for 30 min. Media was replaced and the plate was scanned with a florescent plate reader at excitation 490 nm and emission 515 nm. For (B), protein lysates were separated using SDS-PAGE before immunoblotting with the Mitoprofile Total OXPHOS Antibody Cocktail (Mitosciences). For (C), cells were seeded as for (A), but were stained with 2 µg/mL JC-1 (Life Technologies) for 20 min before the plate was scanned with a florescent plate reader at excitation 490 nm and emission at 522 nm and 605 nm. Data was expressed as a ratio of aggregated JC-1 (605 nm) to monomeric JC-1 (522 nm) as an indication of mitochondrial membrane potential. * denotes statistically different from WT (p<0.01).
(TIF)

Figure S12 HCT116 xenografts from WT and *ADPGK* knockouts. A. Lysates from each tumour were used to confirm the *ADPGK* expression status by western blot (one sample per lane). D. Three different sections from each tumour were stained for either hypoxic areas using pimonidazole (PIMO, green), proliferating areas using bromodeoxyuridine (BrdUrd, brown) or apoptotic areas using the TUNEL assay (red). One example for each group is displayed. PIMO and BrdUrd images are taken in 10× objective magnification. The TUNEL image was taken at 40× objective magnification and represents the region marked with the black box. For bromodeoxyuridine staining, peroxidase block (DAKO Dual Endogenous Enzyme Block) was performed for 10 min at RT before antigen retrieval. Blocking was carried out using rodent block M (Biocare, CA) for 30 min at RT in a humidified chamber, Horseradish peroxidise conjugated rat anti-bromodeoxyuridine antibody (Serotec, UK) was diluted 1∶25 in HRP-STABILPLUS™ (Serotec, UK) and incubated for 1 h at RT in a humidified chamber. Substrate-chromogen DAB (EnVision™ Detection Systems Peroxidase/DAB, DAKO, Denmark) was applied for 8–10 min before being rinsed off. TUNEL assay was performed using the Apoptag® Red In Situ Apoptosis Detection kit (Chemicon International Inc.).
(TIF)

Table S1 HCT116 microarray: top 50 probe sets in order of p values.
(DOCX)

Table S2 Karyotypes of HCT116 WT cells and ADPGK knockout clones.
(DOCX)

Table S3 H460 microarray: top 150 probe sets in order of p values.
(DOCX)

Table S4 Gene ontology (GO) analysis of potential differentially expressed genes in ADPGK knockout clone H460 IIE5 using DAVID.
(DOCX)
