# Supplementary figures and images for: Zinc Finger Nuclease Mediated Knockout of ADP-Dependent Glucokinase in Cancer Cell Lines: Effects on Cell Survival and Mitochondrial Oxidative Metabolism
Source: PLoS One. 2013 Jun 14;8(6):e65267. doi: 10.1371/journal.pone.0065267 (PMC3683018; doi:10.1371/journal.pone.0065267)

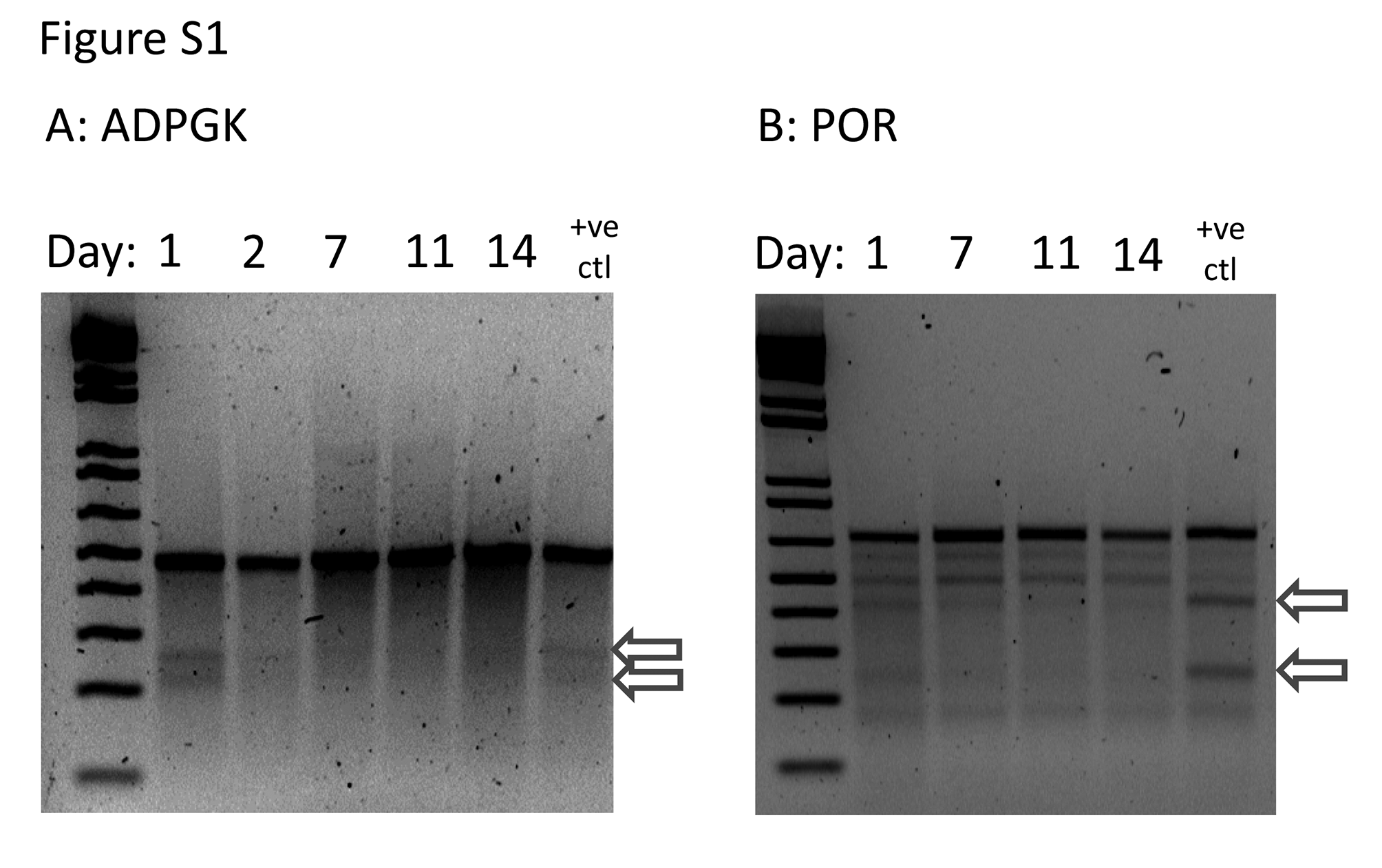

Supplement: Figure S1 — Changes in mutation frequency in HCT116 pools during growth after transfection with ZFNs. HCT116 cells were co-transfected with a GFP plasmid and with plasmid pairs for ADPGK ZFNs (A) and POR ZFNs (B) by nucleofection, and 24 h later 25% of the cells with the brightest GFP fluorescence were sorted. Genomic DNA was prepared on the indicated day after transfection (day 1 sample obtained immediately after sorting), and the mutation frequency was assessed using the Surveyor mutation detection assay. For ADPGK the positive control is a reference DNA sample from ADPGK ZFN-treated K562 cells, provided by Sigma Aldrich, showing the predicted Surveyor products (256 and 217 bp, arrows). For POR the positive control is from a POR null clone (Hko3) with two mutant alleles (a M263L missense mutation and a one-bp insertion) at the ZFN target site that gives predicted Surveyor products of 417 bp and 248 bp (arrows). (TIF) [file pone.0065267.s001.tif]

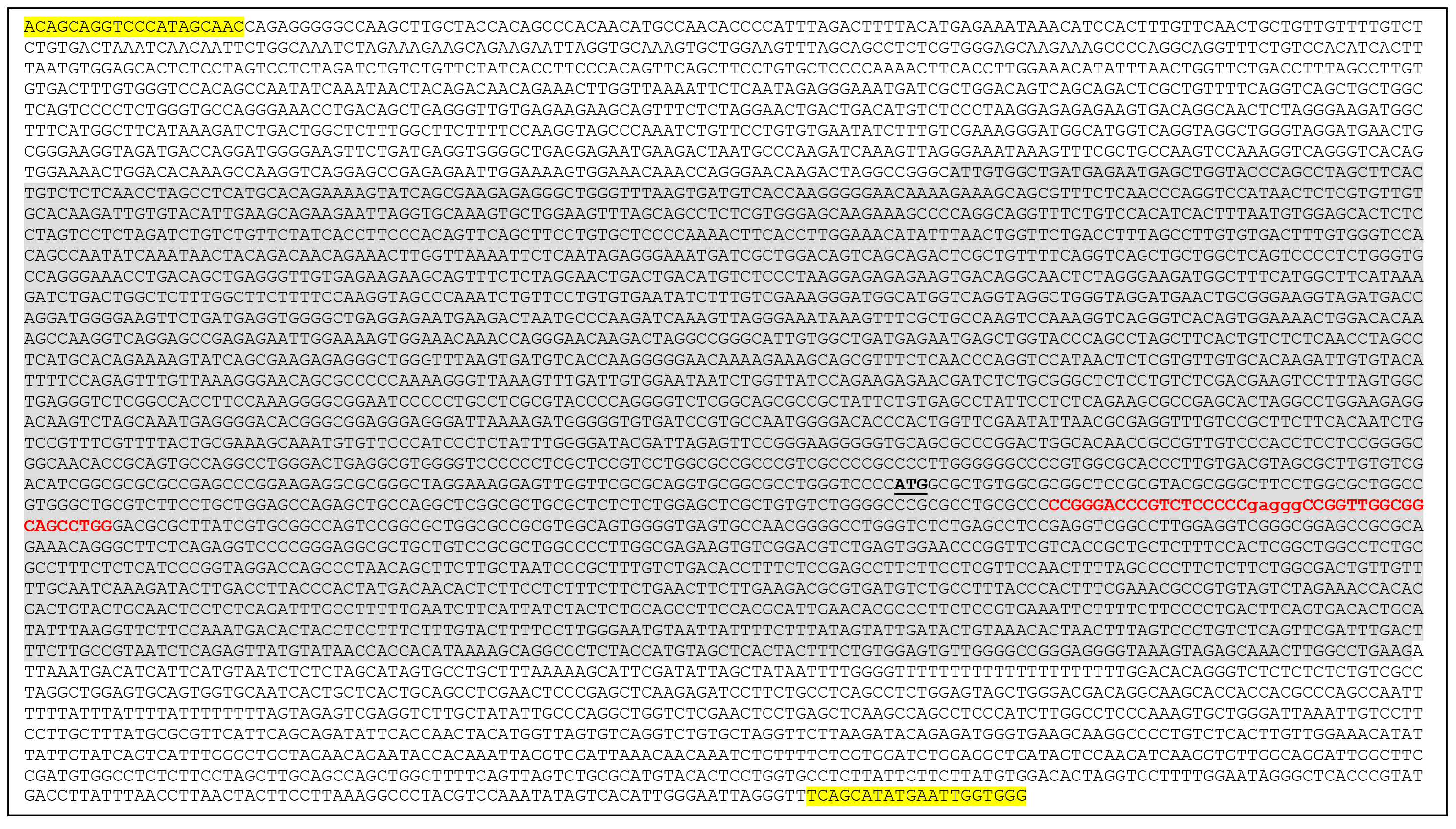

Supplement: Figure S2 — Characterisation of the large ADPGK deletion in HCT116 clone C3. Yellow marks the binding site for primer pair ADPGK_P2. Grey marks the WT sequence deleted in HCT116 C3. The ADPGK start codon ATG is underlined and the ZFN recognition site is marked in red (with cutting site in lower case). (TIF) [file pone.0065267.s002.tif]

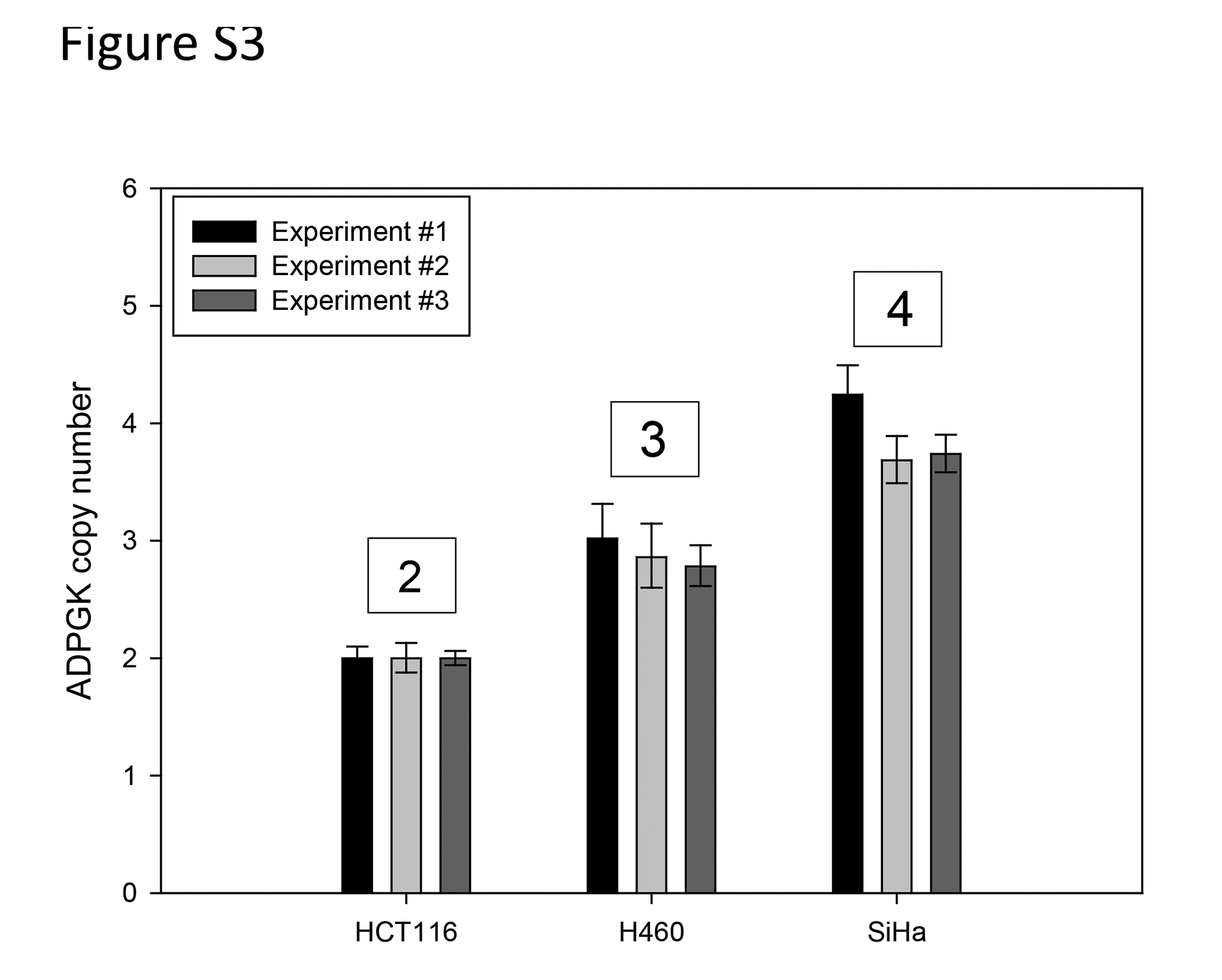

Supplement: Figure S3 — ADPGK copy number of HCT116, H460 and SiHa. Copy number was determined via qPCR with HCT116 as calibrator with known copy number of 2 (according to Sanger Institute, cancer genome project). Primers amplify a 175 bp sequence located within exon 7 of the ADPGK gene. Error bars show the SEM for four technical replicates. Boxed numbers indicate copy number identified by the Sanger Institute (www.sanger.ac.uk/cgi-bin/genetics/CGP/cghviewer/CghViewer.cgi). All cell lines were obtained from the American Type Culture Collection (ATCC), VA. (TIF) [file pone.0065267.s003.tif]

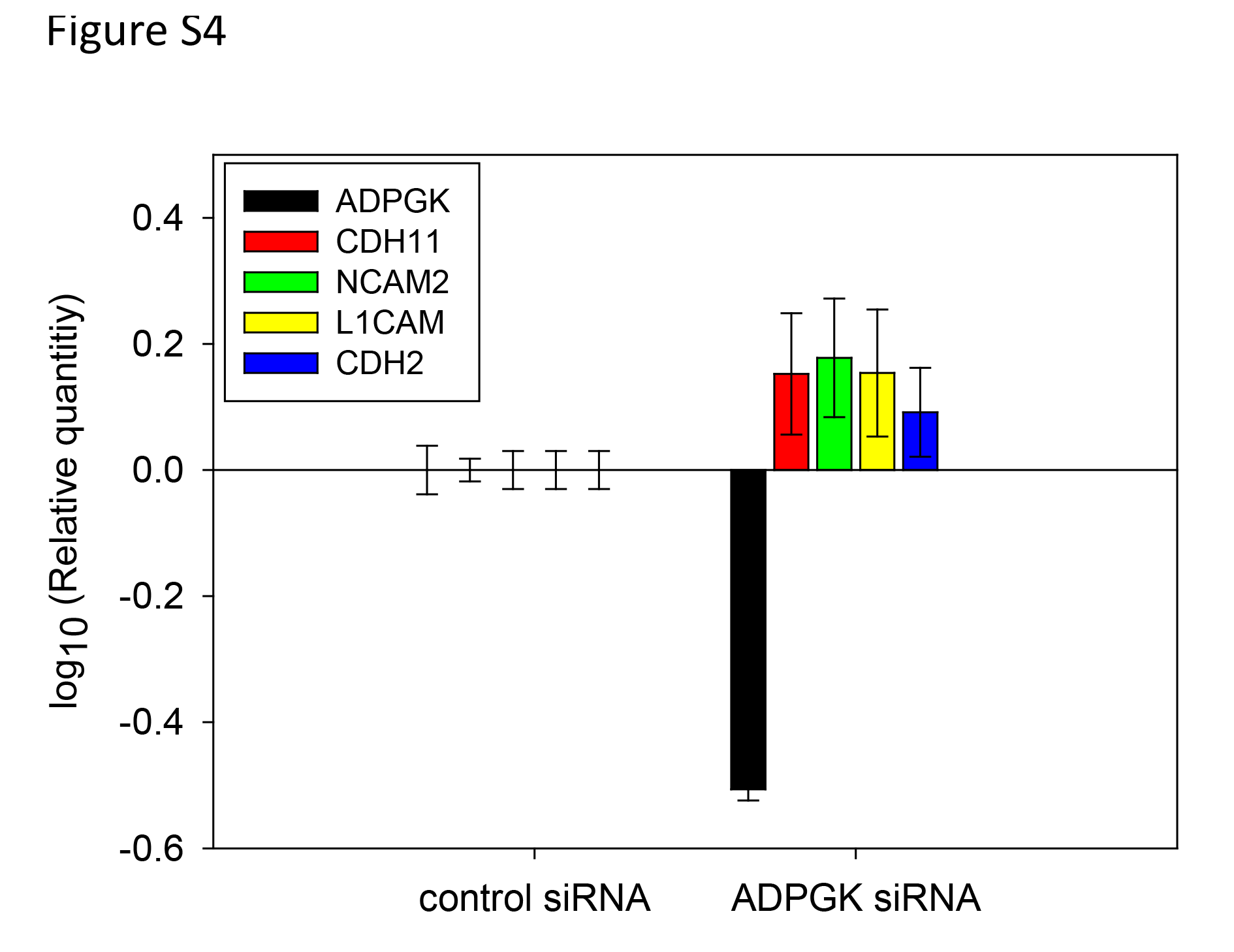

Supplement: Figure S4 — No decreased expression of cell adhesion molecules when ADPGK is knocked down in H460. RNA from three separate experiments was isolated one day after transfection (RNAiMAXTM, Invitrogen, CA) with both control siRNA and ADPGK siRNA (Invitrogen, CA). RNA was transcribed into cDNA and analysed by qPCR. All values are normalised against 18S rRNA, and error bars represent the standard error of three biological replicates each. (TIF) [file pone.0065267.s004.tif]

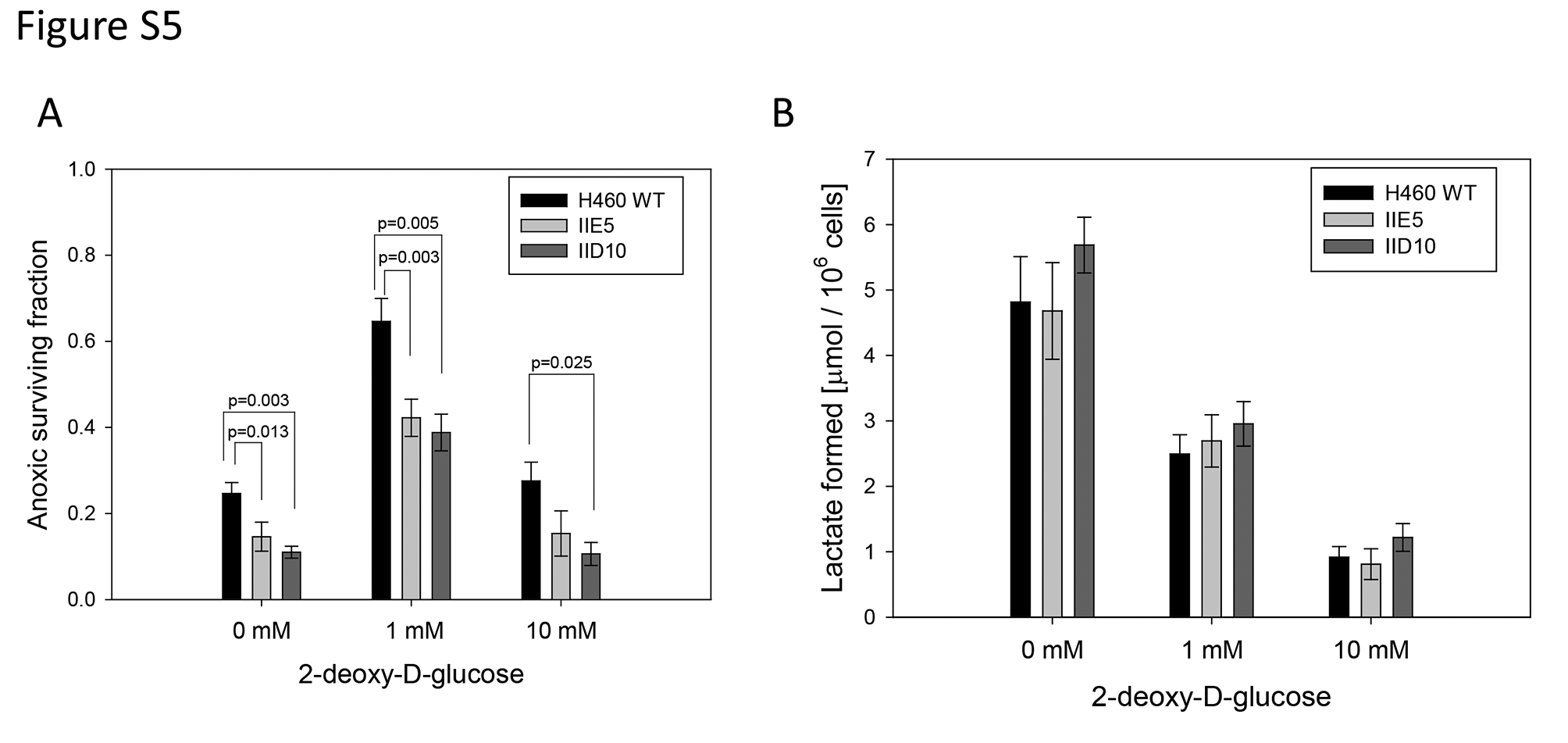

Supplement: Figure S5 — ADPGK KO reduces anoxic cell survival but not lactate formation in H460, and 2-deoxy-D-glucose (2DG) has no effect on these parameters. H460 cells (WT, KO clone IIE5 and IID10) were plated in an anoxic chamber for 2 h before being treated with 2 concentrations of 2DG (1 mM, 10 mM, Sigma-Aldrich, MO) or saline only for 4 h. Graphs show results from 3 independent experiments with 3 experimental replicates each. A. Anoxic surviving fraction measured by clonogenic assay after exposure to 6 h anoxia. B. Lactate formation measured in culture medium after 4 h exposure to saline or 2DG. (TIF) [file pone.0065267.s005.tif]

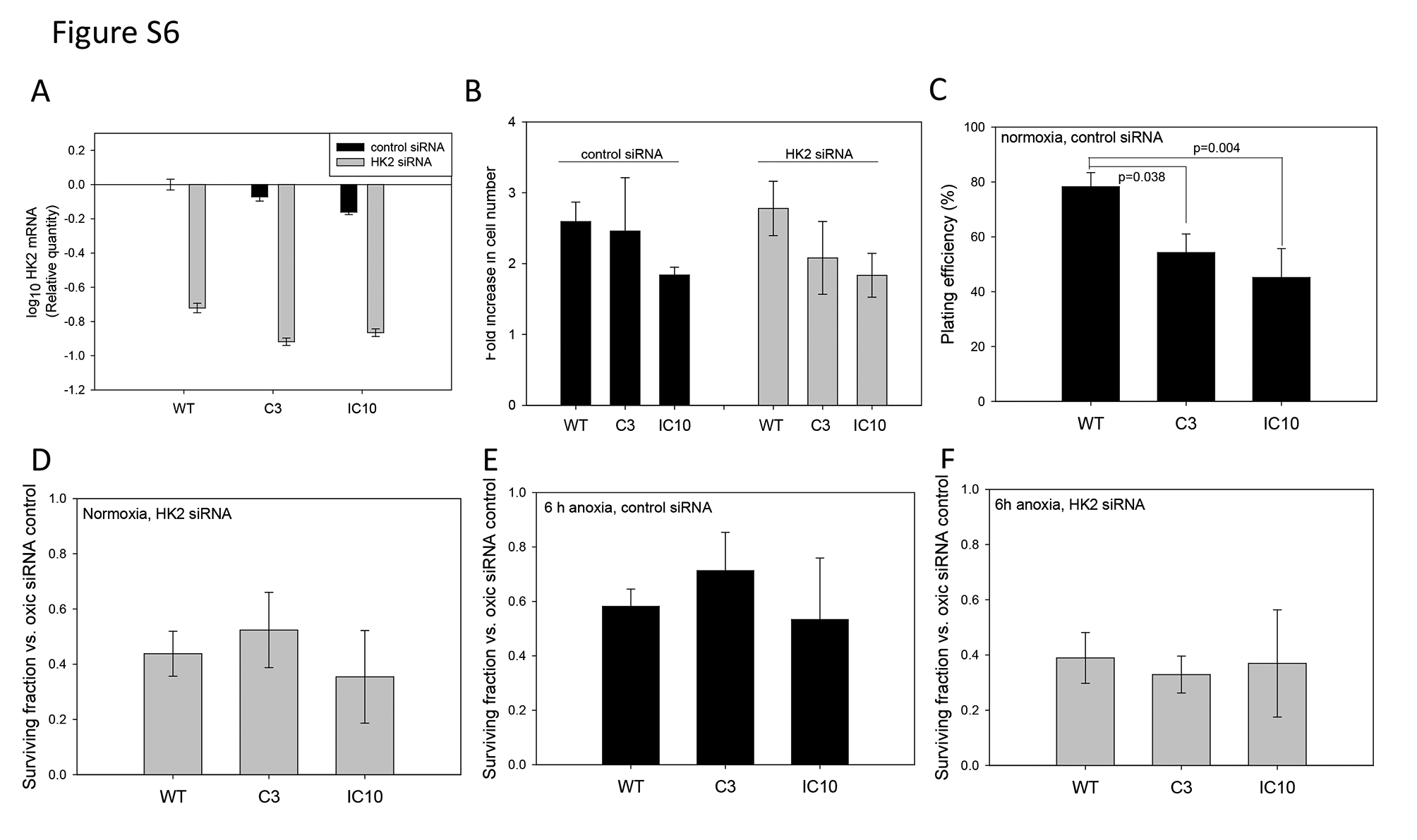

Supplement: Figure S6 — Knockout of ADPGK in HCT116 does not affect clonogenic survival under anoxia and when HK2 is knocked down. HK2 was knocked down by siRNA in two independent experiments, combined here, each of which included three biological replicates for WT and two for each KO clone. (A) HK2 mRNA by qPCR one day after siRNA transfection. (B). Cell number two days after siRNA transfection, at which time cells were replated for clonogenic assay. (C) Plating efficiencies of two days after transfection with control siRNA. (D) Effect of HK2 siRNA on clonogenic surviving fraction two days after transfection, relative to cells transfected with control siRNA. (E) Effect of 6 h anoxia on clonogenic surviving fraction, relative to oxic controls, determined by plating cells in an anoxic chamber two days after siRNA transfection and transferring to an aerobic incubator 6 h later. (F) Effect of HK2 siRNA on clonogenic surviving fraction after exposure to 6 h anoxia, relative to an equivalent anoxic exposure after control siRNA. (TIF) [file pone.0065267.s006.tif]

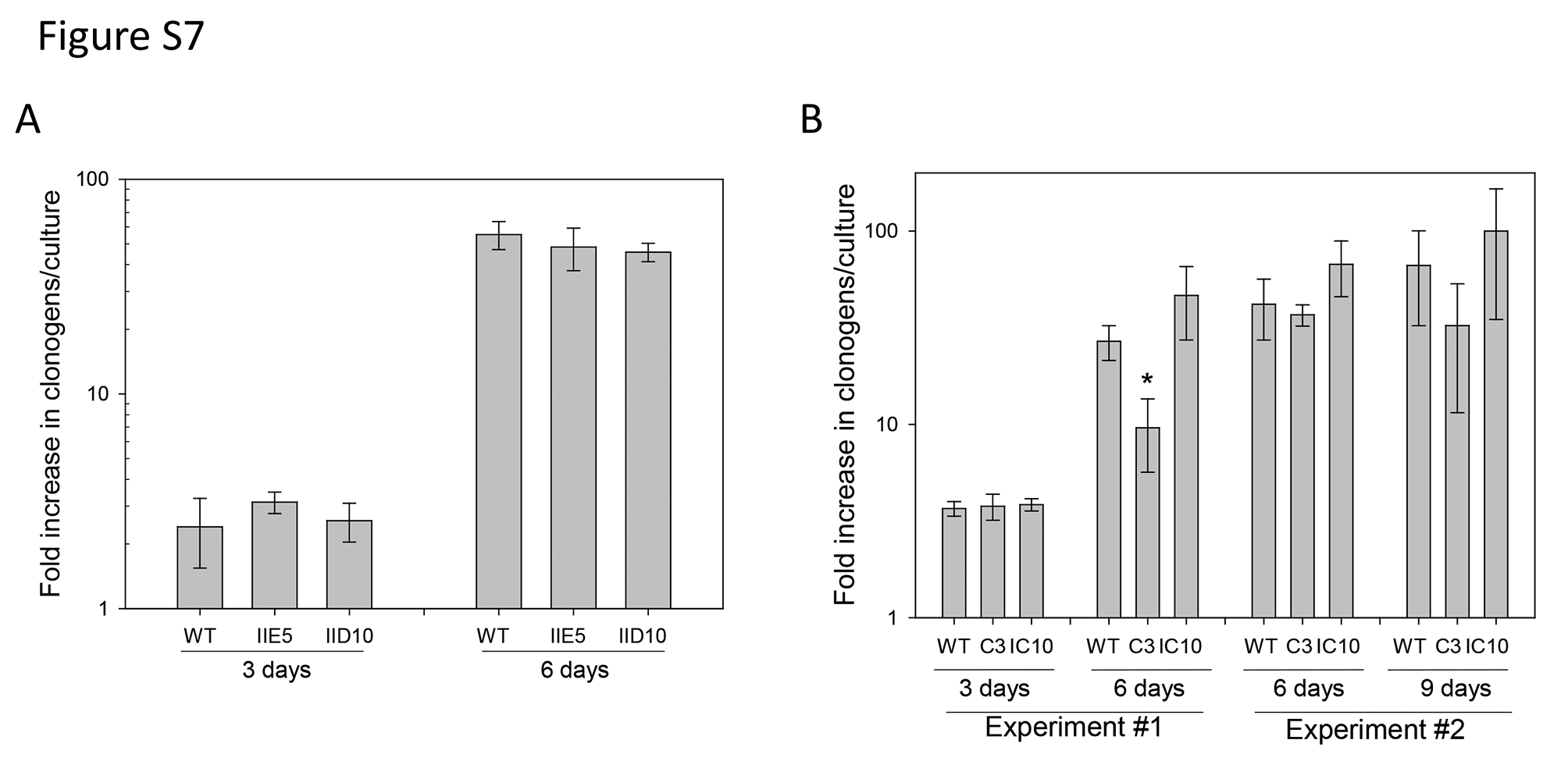

Supplement: Figure S7 — Knockout of ADPGK in H460 (A) and HCT116 (B) does not affect cell growth or clonogenic survival under chronic hypoxia. Cells were seeded at 20,000, 1000 or 200 cells/well into 24-well plates and exposed to 3, 6 or 9 days of hypoxia (0.2% oxygen in gas phase), respectively. Cell number was measured using a Beckman Coulter counter and cells were re-plated for 10 days to measure clonogenic survival. Asterisks indicate significance (p<0.05) compared to WT. For HCT116 cell lines, two separate experiments were performed. Hypoxia (0.2% oxygen 5% CO2/N2) was achieved with an anaerobic glove box system and an oxygen controller (Coy Laboratory Products, Inc.). For long-term exposure, plates were partially enclosed in plastic bags containing trays of water to maximise humidity. (TIF) [file pone.0065267.s007.tif]

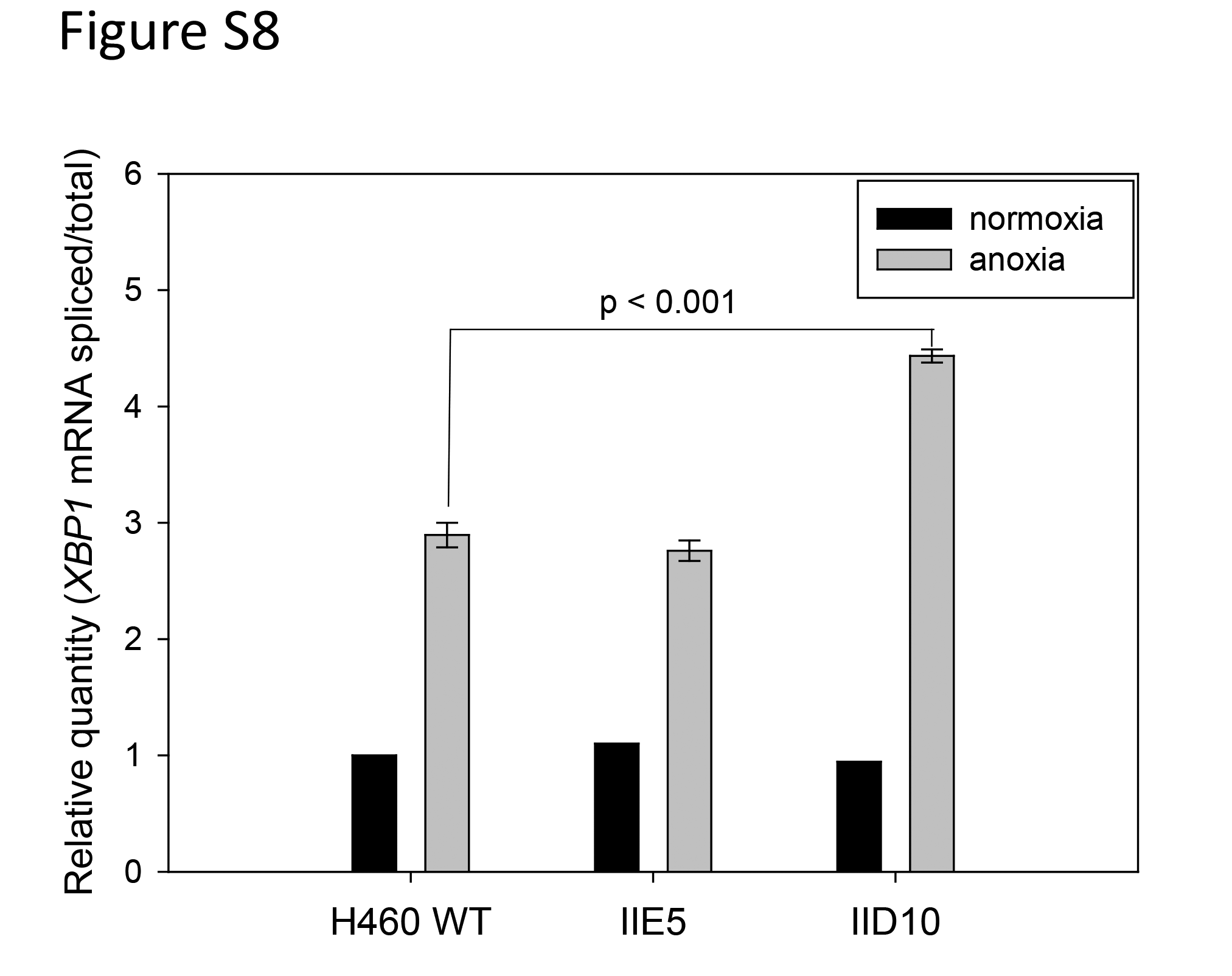

Supplement: Figure S8 — XBP1 splicing under anoxia in H460 cell lines. RNA from H460 WT and two ADPGK-null clones was extracted after exposure to either 6-h normoxia (1 culture each) or anoxia (3 cultures each). RNA was transcribed into cDNA and analysed by qPCR. Two primer pairs, one reporting on total XBP1 (GGCATCCTGGCTTGCCTCCA; GCCCCCTCAGCAGGTGTTCC) and one reporting on spliced XBP1 (CGCTTGGGGATGGATGCCCTG; CCTGCACCTGCTGCGGACT) were used. All values were normalised against 18S rRNA, and the ratio between spliced and total XBP1 was calculated. Error bars represent standard error. (TIF) [file pone.0065267.s008.tif]

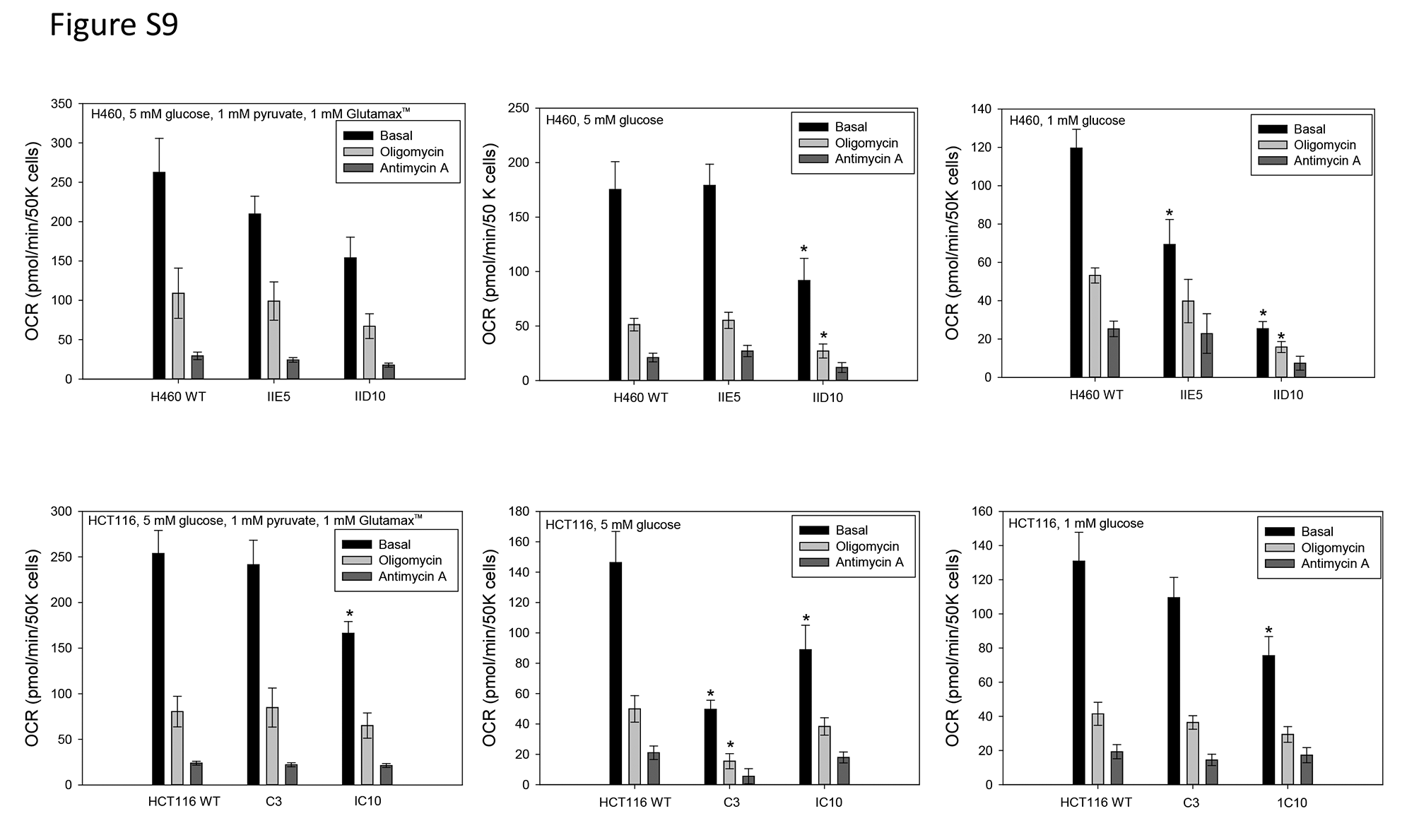

Supplement: Figure S9 — Inhibition of oxygen consumption by oligomycin and antimycin. Oxygen consumption rate (OCR) in ADPGK-null clones and parental (WT) cell lines in the indicated culture media, determined using a Seahorse XF Analyser, and normalised to 50,000 cells. Values are mean and errors are SEM for five biological replicates, and are derived from two timepoints for basal conditions and three measurements after addition of oligomycin (1 µM) and antimycin A (1 µM). Asterisks indicate significance (p<0.05) of differences from the respective WT. (TIF) [file pone.0065267.s009.tif]

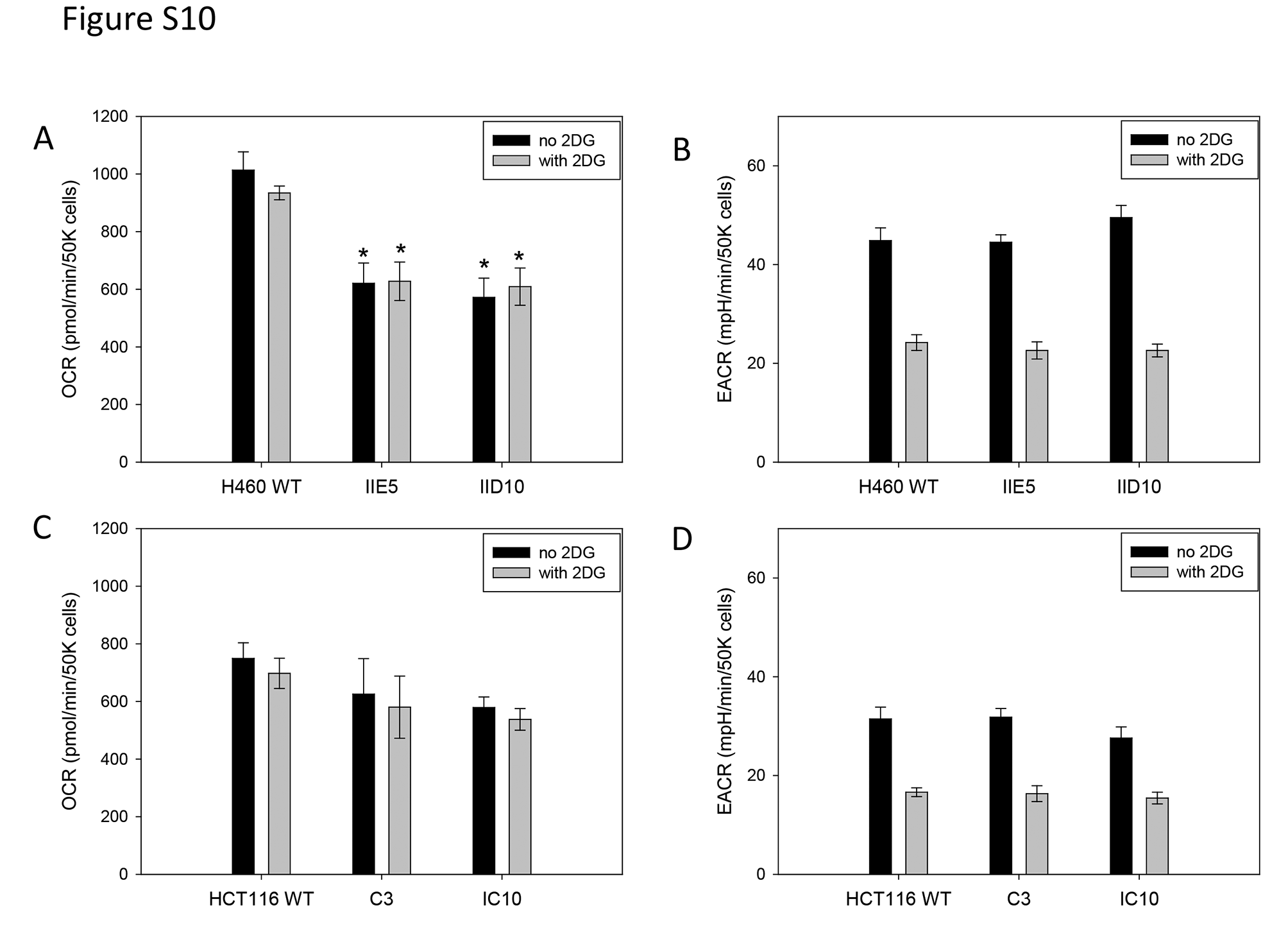

Supplement: Figure S10 — Inhibition of glycolysis by 2-deoxy-D-glucose (2DG). Five replicate cultures of each cell line were seeded in complete medium (5 mM glucose, 1 mM pyruvate, 1 mM glutamate), and EACR and OCR were measured with a Seahorse XF Analyser. After addition of 100 mM 2DG (Sigma-Aldrich) measurements were repeated. Asterisks indicate significance (p<0.05) of differences from the respective WT. (TIF) [file pone.0065267.s010.tif]

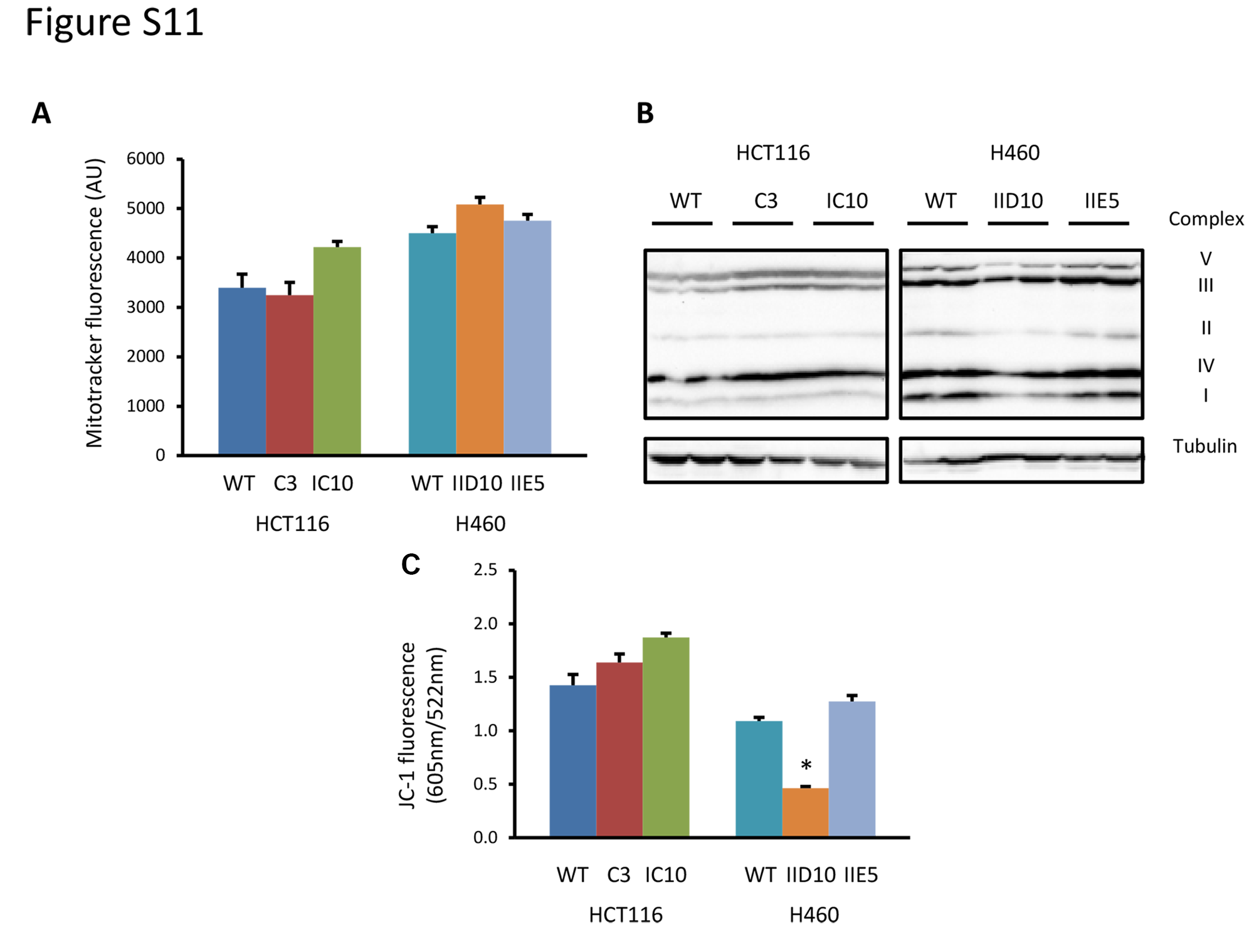

Supplement: Figure S11 — Knockout of ADPGK in H460 and HCT116 has no effect on mitochondrial mass (A), but has clone-dependent effects on electron transport chain subunit expression (B) and mitochondrial membrane potential (C). For (A), cells were seeded at 25,000 cells per well in black, clear bottom 96 well plates. 24 hours later, cells were stained with 50 nM Mitotracker Green FM (Life Technologies) for 30 min. Media was replaced and the plate was scanned with a florescent plate reader at excitation 490 nm and emission 515 nm. For (B), protein lysates were separated using SDS-PAGE before immunoblotting with the Mitoprofile Total OXPHOS Antibody Cocktail (Mitosciences). For (C), cells were seeded as for (A), but were stained with 2 µg/mL JC-1 (Life Technologies) for 20 min before the plate was scanned with a florescent plate reader at excitation 490 nm and emission at 522 nm and 605 nm. Data was expressed as a ratio of aggregated JC-1 (605 nm) to monomeric JC-1 (522 nm) as an indication of mitochondrial membrane potential. * denotes statistically different from WT (p<0.01). (TIF) [file pone.0065267.s011.tif]

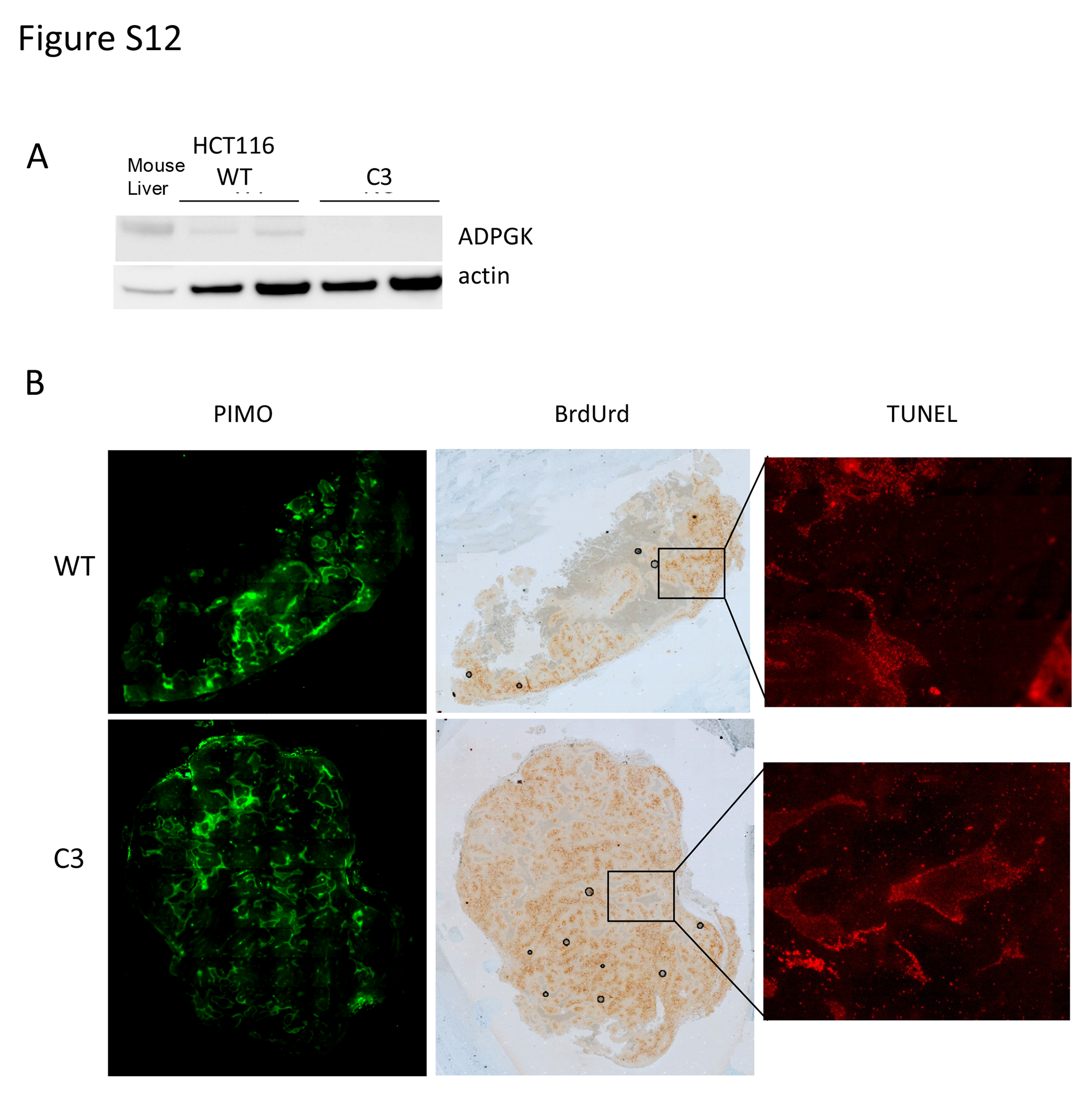

Supplement: Figure S12 — HCT116 xenografts from WT and ADPGK knockouts. A. Lysates from each tumour were used to confirm the ADPGK expression status by western blot (one sample per lane). D. Three different sections from each tumour were stained for either hypoxic areas using pimonidazole (PIMO, green), proliferating areas using bromodeoxyuridine (BrdUrd, brown) or apoptotic areas using the TUNEL assay (red). One example for each group is displayed. PIMO and BrdUrd images are taken in 10× objective magnification. The TUNEL image was taken at 40× objective magnification and represents the region marked with the black box. For bromodeoxyuridine staining, peroxidase block (DAKO Dual Endogenous Enzyme Block) was performed for 10 min at RT before antigen retrieval. Blocking was carried out using rodent block M (Biocare, CA) for 30 min at RT in a humidified chamber, Horseradish peroxidise conjugated rat anti-bromodeoxyuridine antibody (Serotec, UK) was diluted 1∶25 in HRP-STABILPLUS™ (Serotec, UK) and incubated for 1 h at RT in a humidified chamber. Substrate-chromogen DAB (EnVision™ Detection Systems Peroxidase/DAB, DAKO, Denmark) was applied for 8–10 min before being rinsed off. TUNEL assay was performed using the Apoptag® Red In Situ Apoptosis Detection kit (Chemicon International Inc.). (TIF) [file pone.0065267.s012.tif]
