# Supplementary material for: Zinc Finger Nuclease Mediated Knockout of ADP-Dependent Glucokinase in Cancer Cell Lines: Effects on Cell Survival and Mitochondrial Oxidative Metabolism
Source: PLoS One. 2013 Jun 14;8(6):e65267. doi: 10.1371/journal.pone.0065267 (PMC3683018; doi:10.1371/journal.pone.0065267)
Supplement: Table S1 — HCT116 microarray: top 50 probe sets in order of p values. (DOCX) [file pone.0065267.s013.docx]

Table S1: Top 50 probe sets ranked for difference between HCT116 parental and *ADPGK* knockout clone HCT116 C3.

| **ProbeID** | **GenBank** | **Symbol** | **Fold change^a^** | **Name** | **Chr** |
| --- | --- | --- | --- | --- | --- |
| 7998927 | NA ^b^ | NA | 6.00 | NA | 16 |
| 7970404 | NM_130785 | TPTE2 | 0.20 | transmembrane phosphoinositide 3-phosphatase and tensin homolog 2 | 13 |
| 8176375 | NM_001008 | RPS4Y1 | 0.10 | ribosomal protein S4, Y-linked 1 | Y |
| 8176719 | NM_004681 | EIF1AY | 0.20 | eukaryotic translation initiation factor 1A, Y-linked | Y |
| 8177137 | NM_007125 | UTY | 0.38 | ubiquitously transcribed tetratricopeptide repeat gene, Y-linked | Y |
| 7990231 | NR_023318 | ADPGK | 0.33 | ADP-dependent glucokinase | 15 |
| 8176624 | NM_001122665 | DDX3Y | 0.12 | DEAD (Asp-Glu-Ala-Asp) box polypeptide 3, Y-linked | Y |
| 7960865 | NM_006931 | SLC2A3 | 0.45 | solute carrier family 2 (facilitated glucose transporter), member 3 | 12 |
| 8176578 | NM_004654 | USP9Y | 0.50 | ubiquitin specific peptidase 9, Y-linked | Y |
| 8098246 | NM_007193 | ANXA10 | 0.59 | annexin A10 | 4 |
| 7934161 | NM_005041 | PRF1 | 0.45 | perforin 1 (pore forming protein) | 10 |
| 7931930 | NM_006257 | PRKCQ | 2.50 | protein kinase C, theta | 10 |
| 8156321 | NM_003177 | SYK | 1.80 | spleen tyrosine kinase | 9 |
| 7987892 | NM_022473 | ZFP106 | 0.59 | zinc finger protein 106 homolog (mouse) | 15 |
| 8059580 | NM_139072 | DNER | 0.59 | delta/notch-like EGF repeat containing | 2 |
| 8091972 | NM_001105078 | MECOM | 1.80 | MDS1 and EVI1 complex locus | 3 |
| 7976523 | NM_012468 | TCL6 | 1.60 | T-cell leukemia/lymphoma 6 | 14 |
| 8176709 | NM_032576 | CYorf15B | 0.53 | chromosome Y open reading frame 15B | Y |
| 7896589 | --- | NA | 1.70 | NA | - |
| 8176698 | NM_001005852 | CYorf15A | 0.45 | chromosome Y open reading frame 15A | Y |
| 7892859 | --- | NA | 2.10 | NA | - |
| 8176484 | NM_003308 | TSPY1 | 2.40 | testis specific protein, Y-linked 1 | Y |
| 8155508 | NA | NA | 0.67 | NA | 9 |
| 8058388 | NA | NA | 4.60 | NA | 2 |
| 8022572 | NM_080597 | OSBPL1A | 0.53 | oxysterol binding protein-like 1A | 18 |
| 8033780 | NM_024106 | ZNF426 | 0.27 | zinc finger protein 426 | 19 |
| 7974425 | NM_015589 | SAMD4A | 0.67 | sterile alpha motif domain containing 4A | 14 |
| 8082745 | NM_178445 | CCRL1 | 0.63 | chemokine (C-C motif) receptor-like 1 | 3 |
| 7922343 | NM_003326 | TNFSF4 | 0.50 | tumor necrosis factor (ligand) superfamily, member 4 | 1 |
| 7955613 | NM_005556 | KRT7 | 0.53 | keratin 7 | 12 |
| 8175871 | NM_000425 | L1CAM | 0.59 | L1 cell adhesion molecule | X |
| 7960370 | NM_032680 | EFCAB4B | 0.67 | EF-hand calcium binding domain 4B | 12 |
| 7901720 | NM_006252 | PRKAA2 | 0.37 | protein kinase, AMP-activated, alpha 2 catalytic subunit | 1 |
| 7908867 | NM_138391 | TMEM183A | 0.67 | transmembrane protein 183A | 1 |
| 8093320 | NM_182524 | ZNF595 | 0.36 | zinc finger protein 595 | 4 |
| 8112202 | NM_006622 | PLK2 | 0.71 | polo-like kinase 2 (Drosophila) | 5 |
| 7895142 | --- | NA | 2.40 | NA | - |
| 8007118 | ENST00000318329 | NA | 1.50 | NA | 17 |
| 7906954 | NM_002585 | PBX1 | 0.59 | pre-B-cell leukemia homeobox 1 | 1 |
| 7966379 | NM_000432 | MYL2 | 0.67 | myosin, light chain 2, regulatory, cardiac, slow | 12 |
| 7971864 | ENST00000410801 | NA | 1.80 | NA | 13 |
| 7894553 | --- | NA | 0.45 | NA | - |
| 7895120 | --- | NA | 0.59 | NA | - |
| 7894900 | --- | NA | 0.53 | NA | - |
| 7893474 | --- | NA | 0.71 | NA | - |
| 8157696 | NM_001001923 | OR5C1 | 1.50 | olfactory receptor, family 5, subfamily C, member 1 | 9 |
| 8166469 | NR_027783 | SAT1 | 0.56 | spermidine/spermine N1-acetyltransferase 1 | X |
| 8177232 | NM_001146705 | KDM5D | 0.53 | lysine (K)-specific demethylase 5D | Y |
| 7893093 | --- | NA | 0.48 | NA | - |
| 8091402 | NM_138786 | TM4SF18 | 0.40 | transmembrane 4 L six family member 18 | 3 |

^a^ Ratio of intensity for knockout line/parental line.  ^b^ NA – not available.
