# Supplementary material for: Zinc Finger Nuclease Mediated Knockout of ADP-Dependent Glucokinase in Cancer Cell Lines: Effects on Cell Survival and Mitochondrial Oxidative Metabolism
Source: PLoS One. 2013 Jun 14;8(6):e65267. doi: 10.1371/journal.pone.0065267 (PMC3683018; doi:10.1371/journal.pone.0065267)

Table S2. Metaphase G-banding karyotypes of HCT116 parental cells and the *ADPGK* knockout clones HCT116 C3 and HCT116 1C10.

Karyotype A:

Karyotype B:

**Karyotype B:**

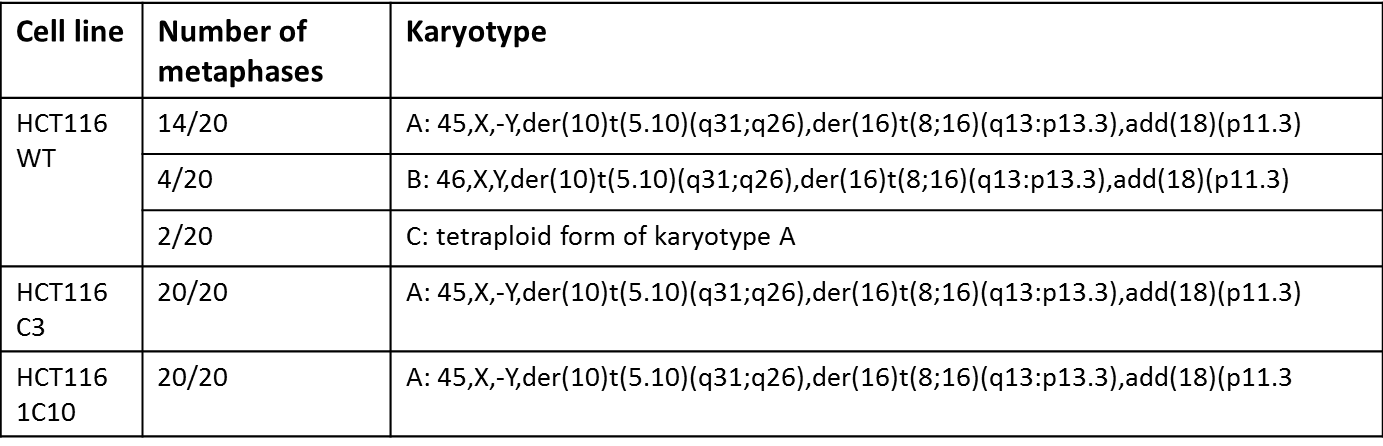

Supplement: Table S2 — Karyotypes of HCT116 WT cells and ADPGK knockout clones. (DOCX) [file pone.0065267.s014.docx]
