# Supplementary material for: Zinc Finger Nuclease Mediated Knockout of ADP-Dependent Glucokinase in Cancer Cell Lines: Effects on Cell Survival and Mitochondrial Oxidative Metabolism
Source: PLoS One. 2013 Jun 14;8(6):e65267. doi: 10.1371/journal.pone.0065267 (PMC3683018; doi:10.1371/journal.pone.0065267)
Supplement: Table S3 — H460 microarray: top 150 probe sets in order of p values. (DOCX) [file pone.0065267.s015.docx]

Table S3: Top 150 probe sets ranked for difference between HCT116 parental and *ADPGK* knockout clone HCT116 C3.

| **ProbeID** | **GeneBank** | **Symbol** | **Fold change^a^** | **Name** |
| --- | --- | --- | --- | --- |
| 8001800 | NM_001797 | CDH11 | 150.7 | cadherin 11, type 2, OB-cadherin (osteoblast) |
| 8059642 | NM_152527 | SLC16A14 | 23.3 | solute carrier family 16, member 14 (monocarboxylic acid transporter 14) |
| 8067985 | NM_004540 | NCAM2 | 9.6 | neural cell adhesion molecule 2 |
| 7954899 | NM_001843 | CNTN1 | 7.6 | contactin 1 |
| 8123936 | NM_001142393 | NEDD9 | 5.6 | neural precursor cell expressed, developmentally down-regulated 9 |
| 8126784 | NM_005084 | PLA2G7 | 15.1 | phospholipase A2, group VII (platelet-activating factor acetylhydrolase, plasma) |
| 8173917 | NM_004538 | NAP1L3 | 7.3 | nucleosome assembly protein 1-like 3 |
| 8177222 | NM_013230 | CD24 | 21.5 | CD24 molecule |
| 7961710 | NM_005691 | ABCC9 | 30.1 | ATP-binding cassette, sub-family C (CFTR/MRP), member 9 |
| 8089145 | NM_015429 | ABI3BP | 0.2 | ABI family, member 3 (NESH) binding protein |
| 8105191 | NM_024615 | PARP8 | 5.0 | poly (ADP-ribose) polymerase family, member 8 |
| 7936968 | NM_003474 | ADAM12 | 0.2 | ADAM metallopeptidase domain 12 |
| 8023822 | NM_182511 | CBLN2 | 0.2 | cerebellin 2 precursor |
| 8094751 | NM_017581 | CHRNA9 | 0.1 | cholinergic receptor, nicotinic, alpha 9 |
| 8114287 | NM_004598 | SPOCK1 | 5.7 | sparc/osteonectin, cwcv and kazal-like domains proteoglycan (testican) 1 |
| 8160670 | NM_004925 | AQP3 | 4.0 | aquaporin 3 (Gill blood group) |
| 8052872 | NM_003236 | TGFA | 0.1 | transforming growth factor, alpha |
| 7938951 | NM_213599 | ANO5 | 3.9 | anoctamin 5 |
| 8171229 | NM_004650 | PNPLA4 | 9.7 | patatin-like phospholipase domain containing 4 |
| 8067652 | NM_001958 | EEF1A2 | 4.0 | eukaryotic translation elongation factor 1 alpha 2 |
| 8134339 | NM_015068 | PEG10 | 4.1 | paternally expressed 10 |
| 7946903 | NM_153676 | USH1C | 5.5 | Usher syndrome 1C (autosomal recessive, severe) |
| 8152522 | NM_006209 | ENPP2 | 0.3 | ectonucleotide pyrophosphatase/phosphodiesterase 2 |
| 8081590 | NM_001134439 | PHLDB2 | 0.1 | pleckstrin homology-like domain, family B, member 2 |
| 7970404 | NM_130785 | TPTE2 | 3.4 | transmembrane phosphoinositide 3-phosphatase and tensin homolog 2 |
| 8157487 | NM_002581 | PAPPA | 3.4 | pregnancy-associated plasma protein A, pappalysin 1 |
| 8089464 | ENST00000383686 | NA | 0.2 | NA^b^ |
| 8129418 | NM_001135648 | PTPRK | 0.3 | protein tyrosine phosphatase, receptor type, K |
| 7917912 | NM_000110 | DPYD | 0.3 | dihydropyrimidine dehydrogenase |
| 8104663 | NM_004932 | CDH6 | 0.2 | cadherin 6, type 2, K-cadherin (fetal kidney) |
| 8099633 | NM_013261 | PPARGC1A | 3.0 | peroxisome proliferator-activated receptor gamma, coactivator 1 alpha |
| 7974461 | NR_003225 | LGALS3 | 3.1 | lectin, galactoside-binding, soluble, 3 |
| 8059648 | ENST00000362530 | NA | 7.8 | NA |
| 8147132 | NM_000067 | CA2 | 0.3 | carbonic anhydrase II |
| 8073929 | NM_015381 | FAM19A5 | 4.6 | family with sequence similarity 19 (chemokine (C-C motif)-like), member A5 |
| 8022674 | NM_001792 | CDH2 | 4.7 | cadherin 2, type 1, N-cadherin (neuronal) |
| 7981949 | NR_003316 | SNORD116-1 | 2.8 | small nucleolar RNA, C/D box 116-1 |
| 8161460 | NM_033655 | CNTNAP3 | 3.1 | contactin associated protein-like 3 |
| 8088919 | NM_133631 | ROBO1 | 3.0 | roundabout, axon guidance receptor, homolog 1 (Drosophila) |
| 8113103 | NM_001145678 | C5orf36 | 2.9 | chromosome 5 open reading frame 36 |
| 7908488 | NM_002113 | CFHR1 | 3.2 | complement factor H-related 1 |
| 8158671 | NM_000050 | ASS1 | 2.7 | argininosuccinate synthetase 1 |
| 7926506 | NM_201596 | CACNB2 | 3.5 | calcium channel, voltage-dependent, beta 2 subunit |
| 7994131 | NM_002738 | PRKCB | 4.2 | protein kinase C, beta |
| 8112007 | NM_198449 | EMB | 8.5 | embigin homolog (mouse) |
| 7925531 | NM_181690 | AKT3 | 0.4 | v-akt murine thymoma viral oncogene homolog 3 (protein kinase B, gamma) |
| 7968417 | NM_023037 | FRY | 3.8 | furry homolog (Drosophila) |
| 8021614 | NM_080475 | SERPINB11 | 13.2 | serpin peptidase inhibitor, clade B (ovalbumin), member 11 (gene/pseudogene) |
| 8135734 | NM_024913 | C7orf58 | 3.3 | chromosome 7 open reading frame 58 |
| 8173245 | NM_017677 | MTMR8 | 2.6 | myotubularin related protein 8 |
| 8101429 | NM_016619 | PLAC8 | 3.0 | placenta-specific 8 |
| 8106923 | NM_005654 | NR2F1 | 3.1 | nuclear receptor subfamily 2, group F, member 1 |
| 7961702 | NM_004982 | KCNJ8 | 3.4 | potassium inwardly-rectifying channel, subfamily J, member 8 |
| 8033233 | NM_006087 | TUBB4 | 2.6 | tubulin, beta 4 |
| 7893373 | --- | NA | 2.9 | NA |
| 8074716 | NR_003267 | GGT3P | 2.7 | gamma-glutamyltransferase 3 pseudogene |
| 8175444 | NM_004114 | FGF13 | 2.4 | fibroblast growth factor 13 |
| 7959016 | NR_027345 | NCRNA00173 | 0.4 | non-protein coding RNA 173 |
| 8100310 | ENST00000391570 | NA | 2.4 | NA |
| 8144448 | NM_018088 | FAM90A1 | 2.5 | family with sequence similarity 90, member A1 |
| 8108905 | NM_020768 | KCTD16 | 2.4 | potassium channel tetramerisation domain containing 16 |
| 8043197 | NM_003761 | VAMP8 | 2.4 | vesicle-associated membrane protein 8 (endobrevin) |
| 8081548 | NM_015480 | PVRL3 | 0.3 | poliovirus receptor-related 3 |
| 8161044 | NM_003289 | TPM2 | 0.4 | tropomyosin 2 (beta) |
| 7962375 | NM_153026 | PRICKLE1 | 3.1 | prickle homolog 1 (Drosophila) |
| 7975324 | NM_020692 | GALNTL1 | 5.4 | UDP-N-acetyl-alpha-D-galactosamine:polypeptideN-acetylgalactosaminyltransferase-like 1 |
| 8105487 | NM_138453 | RAB3C | 3.6 | RAB3C, member RAS oncogene family |
| 8124604 | NA | NA | 2.7 | NA |
| 7964927 | NM_004616 | TSPAN8 | 5.5 | tetraspanin 8 |
| 8174598 | NM_000640 | IL13RA2 | 2.3 | interleukin 13 receptor, alpha 2 |
| 7951662 | NM_001885 | CRYAB | 4.9 | crystallin, alpha B |
| 7896285 | --- | NA | 0.4 | NA |
| 8124967 | NM_004639 | BAT3 | 0.2 | HLA-B associated transcript 3 |
| 8091780 | NM_001038628 | B3GALNT1 | 2.6 | beta-1,3-N-acetylgalactosaminyltransferase 1 (globoside blood group) |
| 7930380 | NM_016824 | ADD3 | 2.5 | adducin 3 (gamma) |
| 8006415 | NM_015544 | TMEM98 | 3.0 | transmembrane protein 98 |
| 7917276 | NM_012152 | LPAR3 | 0.3 | lysophosphatidic acid receptor 3 |
| 7991234 | NM_005928 | MFGE8 | 2.4 | milk fat globule-EGF factor 8 protein |
| 8158684 | ENST00000458976 | NA | 3.2 | NA |
| 8111255 | NM_006727 | CDH10 | 2.7 | cadherin 10, type 2 (T2-cadherin) |
| 7894664 | --- | NA | 0.5 | NA |
| 7932985 | NM_003873 | NRP1 | 0.3 | neuropilin 1 |
| 8088560 | NM_182920 | ADAMTS9 | 2.6 | ADAM metallopeptidase with thrombospondin type 1 motif, 9 |
| 8092552 | NM_006548 | IGF2BP2 | 0.5 | insulin-like growth factor 2 mRNA binding protein 2 |
| 8113358 | NM_005668 | ST8SIA4 | 0.4 | ST8 alpha-N-acetyl-neuraminide alpha-2,8-sialyltransferase 4 |
| 7938834 | NM_182964 | NAV2 | 2.8 | neuron navigator 2 |
| 8017597 | ENST00000443085 | NA | 2.3 | NA |
| 7920333 | ENST00000362695 | NA | 3.9 | NA |
| 8117106 | NM_182757 | RNF144B | 2.4 | ring finger protein 144B |
| 7946757 | NM_017508 | SOX6 | 2.2 | SRY (sex determining region Y)-box 6 |
| 8171624 | NM_001079858 | GPR64 | 2.4 | G protein-coupled receptor 64 |
| 7909730 | NM_001017425 | KCNK2 | 2.3 | potassium channel, subfamily K, member 2 |
| 7978801 | NM_001113498 | MDGA2 | 0.5 | MAM domain containing glycosylphosphatidylinositol anchor 2 |
| 7895183 | --- | NA | 0.4 | NA |
| 7976080 | NM_003608 | GPR65 | 2.8 | G protein-coupled receptor 65 |
| 8138466 | NM_182762 | MACC1 | 0.3 | metastasis associated in colon cancer 1 |
| 7893397 | --- | NA | 0.3 | NA |
| 7986642 | NR_027407 | GOLGA8DP | 2.1 | golgin A8 family, member D (pseudogene) |
| 8135224 | NM_001085386 | NF-E4 | 0.4 | transcription factor NF-E4 |
| 8093104 | NM_138461 | TM4SF19 | 0.3 | transmembrane 4 L six family member 19 |
| 8105495 | NR_024617 | PART1 | 2.3 | prostate androgen-regulated transcript 1 (non-protein coding) |
| 8112258 | ENST00000365147 | NA | 3.6 | NA |
| 7951178 | NM_004621 | TRPC6 | 2.5 | transient receptor potential cation channel, subfamily C, member 6 |
| 8142687 | NM_005302 | GPR37 | 2.1 | G protein-coupled receptor 37 (endothelin receptor type B-like) |
| 7932826 | NM_020848 | KIAA1462 | 3.0 | KIAA1462 |
| 8056151 | NM_007366 | PLA2R1 | 2.2 | phospholipase A2 receptor 1, 180kDa |
| 8177354 | NM_004081 | DAZ1 | 0.1 | deleted in azoospermia 1 |
| 8083422 | NM_033050 | SUCNR1 | 0.5 | succinate receptor 1 |
| 8092750 | NM_021032 | FGF12 | 2.0 | fibroblast growth factor 12 |
| 7989365 | NM_134260 | RORA | 2.3 | RAR-related orphan receptor A |
| 8108693 | NM_018938 | PCDHB4 | 0.4 | protocadherin beta 4 |
| 8160459 | NM_004432 | ELAVL2 | 2.0 | ELAV (embryonic lethal, abnormal vision, Drosophila)-like 2 (Hu antigen B) |
| 8110932 | NM_003966 | SEMA5A | 2.5 | sema domain, seven thrombospondin repeats (type 1 and type 1-like), transmembrane domain (TM) and short cytoplasmic domain, (semaphorin) 5A |
| 8060963 | NM_003081 | SNAP25 | 0.5 | synaptosomal-associated protein, 25kDa |
| 8100393 | NM_002253 | KDR | 2.1 | kinase insert domain receptor (a type III receptor tyrosine kinase) |
| 8171472 | NM_020665 | TMEM27 | 3.9 | transmembrane protein 27 |
| 7927099 | NR_026827 | LOC84856 | 0.5 | hypothetical LOC84856 |
| 8152512 | NM_002546 | TNFRSF11B | 2.5 | tumor necrosis factor receptor superfamily, member 11b |
| 7894255 | --- | NA | 0.5 | NA |
| 8021685 | NM_001093729 | CCDC102B | 0.4 | coiled-coil domain containing 102B |
| 8101260 | NM_058172 | ANTXR2 | 0.4 | anthrax toxin receptor 2 |
| 8113800 | NM_001999 | FBN2 | 3.1 | fibrillin 2 |
| 8143610 | NM_005435 | ARHGEF5 | 2.1 | Rho guanine nucleotide exchange factor (GEF) 5 |
| 8109773 | NM_001161661 | WWC1 | 0.5 | WW and C2 domain containing 1 |
| 8112469 | BC171739 | GUSBL1 | 3.9 | glucuronidase, beta-like 1 |
| 7996081 | NM_201524 | GPR56 | 2.1 | G protein-coupled receptor 56 |
| 8173261 | NM_018684 | ZC4H2 | 3.7 | zinc finger, C4H2 domain containing |
| 8101675 | NM_004827 | ABCG2 | 2.3 | ATP-binding cassette, sub-family G (WHITE), member 2 |
| 8116418 | NM_005110 | GFPT2 | 0.3 | glutamine-fructose-6-phosphate transaminase 2 |
| 7990231 | NR_023318 | ADPGK | 3.0 | ADP-dependent glucokinase |
| 8175871 | NM_000425 | L1CAM | 2.7 | L1 cell adhesion molecule |
| 8076424 | NM_000106 | CYP2D6 | 0.2 | cytochrome P450, family 2, subfamily D, polypeptide 6 |
| 8080868 | NR_026866 | C3orf49 | 3.0 | chromosome 3 open reading frame 49 |
| 8148040 | NM_052886 | MAL2 | 2.2 | mal, T-cell differentiation protein 2 |
| 8113097 | NM_001145678 | C5orf36 | 2.8 | chromosome 5 open reading frame 36 |
| 8016390 | NM_016429 | COPZ2 | 2.2 | coatomer protein complex, subunit zeta 2 |
| 8001529 | ENST00000360853 | NA | 0.5 | NA |
| 7893985 | --- | NA | 0.4 | NA |
| 7954398 | NM_030572 | C12orf39 | 2.0 | chromosome 12 open reading frame 39 |
| 8089835 | NM_007085 | FSTL1 | 2.6 | follistatin-like 1 |
| 8114119 | NM_015082 | FSTL4 | 3.2 | follistatin-like 4 |
| 7916882 | NM_000329 | RPE65 | 0.4 | retinal pigment epithelium-specific protein 65kDa |
| 7938485 | NM_014632 | MICAL2 | 2.3 | microtubule associated monoxygenase, calponin and LIM domain containing 2 |
| 7904158 | NM_020190 | OLFML3 | 0.2 | olfactomedin-like 3 |
| 7894780 | --- | NA | 1.9 | NA |
| 7908993 | NM_005686 | SOX13 | 0.5 | SRY (sex determining region Y)-box 13 |
| 8102988 | NM_198682 | GYPE | 3.5 | glycophorin E (MNS blood group) |
| 7908481 | NM_021023 | CFHR3 | 1.9 | complement factor H-related 3 |
| 7931951 | NM_001029880 | SFMBT2 | 3.9 | Scm-like with four mbt domains 2 |
| 7981996 | NR_003338 | SNORD116-24 | 2.0 | small nucleolar RNA, C/D box 116-24 |

^a^ Ratio of intensity for knockout line/parental line.  ^b^ NA – not available.
