# Supplementary material for: Zinc Finger Nuclease Mediated Knockout of ADP-Dependent Glucokinase in Cancer Cell Lines: Effects on Cell Survival and Mitochondrial Oxidative Metabolism
Source: PLoS One. 2013 Jun 14;8(6):e65267. doi: 10.1371/journal.pone.0065267 (PMC3683018; doi:10.1371/journal.pone.0065267)
Supplement: Table S4 — Gene ontology (GO) analysis of potential differentially expressed genes in ADPGK knockout clone H460 IIE5 using DAVID. (DOCX) [file pone.0065267.s016.docx]

Table S4. Gene ontology analysis of potential differentially expressed genes in *ADPGK* knockout clone H460 IIE5 using DAVID (Huang et al., 2009)


 ^1^ significance of gene–term enrichment with a modified Fisher’s exact test (EASE score), ^2^ overall enrichment score for the group based on the EASE scores of each term member.
